# EEF1AKMT4-eEF1A2 synergistically facilitates the progression of GBC by promoting ribosomal protein output

**DOI:** 10.1016/j.gendis.2025.101619

**Published:** 2025-04-02

**Authors:** Yun-cheng Li, Qiang Gao, Yong-chang Tang, Zhen-yu Shao, Jia-ming Hu, Zeng-li Liu, An-da Shi, Shao-hui Huang, Yun-fei Xu, Zong-li Zhang, Kang-shuai Li

**Affiliations:** a Department of General Surgery, Qilu Hospital, Cheeloo College of Medicine, Shandong University, Jinan, Shandong 250012, China; b Department of Radiotherapy, Qilu Hospital, Cheeloo College of Medicine, Shandong University, Jinan, Shandong 250012, China; c Department of General Surgery, Qilu Hospital (Qingdao), Cheeloo College of Medicine, Shandong University, Qingdao, Shandong 266035, China

**Keywords:** eEF1A2, EEF1AKMT4, GBC, Lymph node metastasis, Protein synthesis

## Abstract

Gallbladder cancer (GBC) is prone to lymph node metastasis. Lymph node (LN) metastasis is correlated with abysmal patient prognosis, but the underlying mechanism remains elusive. In this study, transcriptome sequencing of 6 paired GBC tumors and metastatic LNs was performed and identified eEF1A2 as key genes associated with GBC LN metastasis. qPCR, Western blotting and immunohistochemistry (IHC) were performed to assess the expression of eEF1A2 and relating proteins in GBC. The function of eEF1A2 and its regulators were demonstrated in different GBC cell lines as well as in xenograft models. Two independent cohorts of GBC patients were used to reveal the clinical significance. The results revealed that eEF1A2 is tightly correlated with lymph node metastasis and poor prognosis in patients with GBC. In two GBC cell lines, eEF1A2 knockdown impaired cell proliferation, migration, and invasion *in vitro* and inhibited tumor growth and lymph node metastasis *in vivo*, whereas overexpression of eEF1A2 promoted these processes. EEF1AKMT4 trimethylates eEF1A2 at K36 site in GBC and is essential for the tumor-promoting effect of eEF1A2. Mechanistically, trimethylation at the K36 site of eEF1A2 increased the GTPase activity of eEF1A2 and enhanced tumor promoting signals including ERK1/2 and AKT by promoting the ribosome total protein synthesis. In conclusion, the evolutionarily conserved EEF1AKMT4-eEF1A2K36me3-ribosome protein synthesis-tumor promoting signals axis acts as a mechanism that promotes GBC progression and may be a potential therapeutic target for GBC lymph node metastasis.

## Introduction

Biliary tract cancer (BTC) is a series of invasive adenocarcinomas that originate from the epithelium of the biliary tract.^1^ According to its anatomical location, it can be divided into gallbladder cancer (GBC) and cholangiocarcinoma.^2^^,^^3^ Gallbladder cancer is the most common cancer of the biliary system, with a high incidence in middle-aged and elderly women.^4^ Patients with gallbladder cancer are prone to lymph node metastases. Lymph node metastasis is an important factor for poor prognosis.^5^ Although extensive studies have been conducted on the etiology and progression mechanism of GBC, the mechanism of lymph node metastasis has not yet been fully elucidated.^6^

In humans, two eukaryotic translation elongation factor 1 (eEF1A) paralogs function as transporters of aminoacyl tRNA to the ribosome during protein translation elongation, namely eEF1A1 and eEF1A2. The eEF1A1 and eEF1A2 genes are evolutionary conserved.^7^ Although they have high sequence similarity, the distribution and expression patterns of eEF1A1 and eEF1A2 in tissues are different. eEF1A1 is expressed in almost all tissues; however, eEF1A2 is mainly expressed in differentiated tissues such as skeletal muscle, heart muscle, and oral mucosa.^8^ In cancer, the expression of eEF1A2 could be reactivated.^9^ High eEF1A2 expression is associated with poor prognosis in pancreatic ductal adenocarcinoma, non-small cell lung cancer, and ovarian cancer.^10^, ^11^, ^12^ The canonical translational prolongation role, which depends on the GTPase activity of eEF1A2, may play a crucial role in promoting tumor progression.^13^ Increasing evidence has also revealed that ectopic expression of eEF1A2 may promote cancer by regulating the JAK/STAT or AKT signaling pathways.^14^^,^^15^ However, detailed molecular details of the function of eEF1A2 and the underlying mechanism of eEF1A2 in tumors remain to be elucidated.

Lysine methylation is a common modification of nucleic acids and proteins, especially histones.^16^ Depending on the number of methyl groups added, lysine methylation can be divided into monomethylation, dimethylation and trimethylation.^17^ Although different methylation states may have different functions in histones,^18^ the methylation states and functions of lysine methylation in non-histone proteins are largely unknown. eEF1A2 is a highly methylated protein and its methylation mainly focuses on K36, K55, K79, K165, and K318. Interestingly, methylation at each site is controlled specifically by one methylase, and eEF1A2 seems to be the only substrate of these enzymes to date.^19^ Methylation of eEF1A2 may play a role in its function, particularly in mRNA translation. The methylation changes in eEF1A2 and its role in cell biology and tumorigenesis have rarely been investigated.

In this study, we first examined transcriptome differences in six paired GBC tumors and metastatic lymph nodes. We found for the first time that eEF1A2 is highly expressed in GBC, and that high eEF1A2 expression is associated with lymph node metastasis and poor prognosis in patients. We also investigated the function, methylation status, and regulation of eEF1A2 K36 in the GBC cells. We demonstrate that the regulation of translation by the EEF1AKMT4-eEF1A2K36me3 axis acts as a mechanism that promotes the progression and lymph node metastasis of GBC and may be a potential therapeutic target for GBC.

## Methods

### Patient and ethical approval

The study has passed the ethical review by the research Ethics Committee of Shandong University Qilu Hospital. Approval number: KYLL-202011-077. We have obtained informed consent from all participants. The patient cohort comprised patients who underwent surgery for gallbladder cancer at the Qilu Hospital of Shandong University from 2017.1 to 2020.12. The patient cohorts were selected according to the following inclusion criteria: (i) patients who underwent radical resection with a clear surgical margin, (ii) patients with available formalin-fixed tumor tissues, follow-up information, and complete medical records, and (iii) patients with no history of other malignancies. Fresh tumor tissues and tumor-adjacent normal tissues were obtained from six pairs of patients with gallbladder cancer. Tumors were classified and staged according to the 8th AJCC/UICC TNM classification system. The study protocols were approved and supervised by the Research Ethics Committee of Qilu Hospital of Shandong University.

### Cells and agents

Human renal epithelial cell line HEK293T, cholangiocarcinoma cell line RBE, and gallbladder cancer cell lines NOZ, GBCSD, SGC996, and OCUG-1 were used in this study. All cell lines were purchased from the Cell Bank of the Chinese Academy of Sciences (Shanghai, China), authenticated using STR analysis, and tested for mycoplasma contamination. RBE, GBC-SD, OCUG-1, and SGC996 cells were cultured in RPMI 1640 (Thermo Fisher Scientific, Waltham, MA, USA), while NOZ and HEK293T cells were cultured in DMEM (Thermo Fisher Scientific). The media for the cell lines were supplemented with 10% fetal bovine serum (Thermo Fisher Scientific), 1% penicillin/streptomycin at 37 °C under 95% air and 5% CO_2_.

The eEF1A2 K36me3 antibody was produced through antigen preparation, animal immunization, antiserum purification, etc., and is authorized to be completed by Daian biotechnology. The reagents and antibodies used were as follows:

**Table:** 

Antibodies	Source	Identifier
eEF1A2	Abcam	ab212172
EEF1AKMT4	Proteintech	Cat No. 15418-1-AP
eEF1A2K36me3	Daian biotechnology	N/A
Puromycin	Sigma	MABE343
AKT	CST	#9272
ERK1/2	Immunoway	YT1625
β-actin	Abcam	ab8226
Chemicals, peptides and chemicals, peptides		
Protein A/G beads	MCE	Cat. No.: HY-K0202
Anti-flag-tag beads	MCE	Cat. No.: HY-K0207
ATPase/GTPase activity assay kit	Abcam	Ab272520
Biological samples		
Human GBC tissue array	Shanghai OUTDO BIOTECH co., ltd.	HGalA145PG01

### Tissue microarray and immunohistochemistry (IHC)

Human gallbladder cancer tissue microarray was purchased from Shanghai OUTDO BIOTECH Co.; Ltd. Immunohistochemistry (IHC)analysis was performed as previously described.^20^ IHC was performed to detect eEF1A2. Briefly, the slides were submerged in EDTA buffer (pH 8.0) for optimal antigen retrieval. The tissue microarray slides were incubated with rabbit antibody anti-eEF1A2 (1:100; Abcam, USA) at 4 °C overnight. Biotin-labeled goat anti-rabbit antibody (Zsbio, Beijing, China) was applied for 30 min at room temperature. Slides were then incubated with horseradish peroxidase-conjugated streptavidin. The peroxidase reaction was developed using 3,3-diaminobenzidine (DAB) solution (Zsbio).

IHC results of the tissue microarray were qualified using Quant Center software as previously reported,^20^^,^^21^ which contains the synthetic score of the staining intensity and the area of each staining. IHC score= (percentage of cells with weak intensity × 1) + (percentage of cells with moderate intensity × 2) + (percentage of cells with strong intensity × 3). The cutoff IHC score was calculated using receiver operating characteristic (ROC) curves. The cohort was divided into subsets with low or high expression of candidate biomarkers using this cutoff.

The IHC results of the gallbladder cancer cohort were evaluated independently by two senior pathologists who were unaware of the clinical information. The IHC results were semi quantitatively scored in a conventional manner based on the staining intensity (0, negative; 1, weak; 2, moderate; 3, strong) and the percentage of positively stained cells (0, 0%; 1, 1%–25%; 2, 25%–50%; 3, 50%–75%; 4, 75%–100%). The final score is the product of the two scores.

### RNA extraction and qPCR

Total RNAs from tissues and cells was extracted according to the manufacturer's protocols using TRIzol reagent (Thermo Fisher). A reverse transcriptase kit (Vazyme, China) was used to synthesize cDNA following the manufacturer's recommendations. Real-time PCR was performed using the SYBR Green Master Mix (Vazyme, China) and a Light Cycler Roche 480 PCR instrument. Comparisons between the groups were performed using the 2^−ΔΔCt^ method. Primers used for qPCR are listed in the following table.

**Table:** 

qPCR primer sequence	Forward	Reverse
eEF1A2	GAAGACCCACATCAACATCGT	CTCCGCATTTGTAGATGAGGTG
eEF1A1	TGTCGTCATTGGACACGTAGA	ACGCTCAGCTTTCAGTTTATCC
CD109	AAGCCAGTGAAAGGAGACGTA	CCAGGGGAAGATAGATCCAGG
CEACAM5	CTGTCCAATGACAACAGGACC	ACGGTAATAGGTGTATGAGGGG
TMEM145	AAAGGTCGTCAGTTGCTCCAC	TTGACCCCAGTAGATGCAGAA
SYT7	TCATCACCGTCAGCCTTAGC	TCTTGTAGCGTTTGCCCAGTT
LAMC3	CCCACCTCGGTCAACATCAC	GAGGCGCTGTAGAACTGGTA
TNS4	AGGACACCAGAACTCCGTTCA	TCTCGGGTGATGTTTGGCTTA
ADORA2B	TGCACTGACTTCTACGGCTG	GGTCCCCGTGACCAAACTT
EEF1AKMT4	AGAGAAAACGGGGCCAAGAG	TTGTTGCTCCCTCACCTCAC
METTL13	CAGGAGGTTGATTACAGTGGC	CTCCATGACTCTAGCCGACA
N6AMT2	CAAAAGCAACAAATTGAGCCAGG	GGCACTCACACATGCGATT
METTL21B	ATCCCGAATCTGAGTCGGAAT	ACTCTCGAAATAATTGCACAGGC
METTL10	TCAGTGCTTGATATTGGAACTGG	GCTGAAGTGGAAAAGATCGCTTG
AKT	ATCGCTTCTTTGCCGGTAT	TCTTGGTCAGGTGGTGTGAT
ERK	TTACTGCGCTTCAGACATGAGA	ATCTGTTTCCATGAGGTCCTGT

### Western blotting

Tissue and total cell proteins were extracted using RIPA lysis buffer (Sparkjade, Shandong, China) with 1% PMSF (Sparkjade, Shandong, China) and 1% phosphatase inhibitor (Solarbio). A total of 30 μg protein was subjected to Western blotting and then separated using 10% SDS-PAGE. SDS-PAGE was then electro-transferred onto polyvinylidene difluoride membranes. Membranes were blocked using 5% BSA and then subjected to incubation with the primary antibody at 4 °C and then secondary antibodies for 1 h. Protein bands were visualized using enhanced chemiluminescence (Millipore) according to the manufacturer's instructions. Quantification of the protein bands was performed using the ImageJ software.

### Transfection and stable cell lines

Knockdown or overexpression of eEF1A2 in GBCSD and SGC996 cells was performed using a lentivirus (Gene Pharma). Other transfections of GBCSD and SGC996 cells were performed using Lipofectamine 2000 (Thermo Fisher Scientific). Stable cell lines were established using 4 μg/mL puromycin. Puromycin-resistant clones were isolated for further culture. The sequences of the siRNAs and shRNAs are listed in following table.

**Table:** 

Name	The target sequence ofshRNAs and siRNA(5′–3′)
sheEF1A2-1	CGCGACTTCATCAAGAACA
sheEF1A2-2	GCGCCTACATCAAGAAGA
shEEF1AKMT4-1	ACTATGCCCAAGCCTATTA
shEEF1AKMT4-2	CTTAGTGCCATTCAGCTCT
siMETTL13-1	GCGGUGACUAUGUCAUUGATT
siMETTL13-2	GGCUUCAGGAGGUUGAUUATT

### CCK8 assay

The CCK8 assay was performed as previously described.^22^ Medium (200 μL of medium containing three thousand cells was seeded into each well of a 96-well plate, and cell proliferation was measured at 6, 24, 48, 72 and 96 h after seeding, according to the manufacturer's protocol. Briefly, cells were incubated with 100 μL of reaction mixture containing 10 μL CCK-8 and 90 μL DMEM for 2 h and then measured at a wavelength of 450 nm.

### Clone formation assay

Clone formation assays were performed as previously described.^23^ One thousand cells were seeded into a six-well plate and then cultured under 5% CO_2_ at 37 °C for 2 weeks (GBCSD) or 4 weeks (SGC996 cells). The colonies were fixed with formalin for 30 min and then stained with 0.1% crystal violet for 15 min.

### Cell invasion assay

Cell invasion was evaluated using transwell chambers coated with matrigel. In brief, a total of 3 × 10^4^ cells were seeded into the upper chamber of the 8.0-μm pore polycarbonate membrane for adhesion with a monolayer of 5% Matrigel. The chambers were then placed in one pore of the 24-well plates containing medium with 20% FBS at the bottom. After incubation at 37 °C for 24 h (RBE cells), the cells migrating to the lower surface of the chamber were fixed with paraformaldehyde and then stained with 0.1% crystal violet solution (Sigma–Aldrich). Three visual fields were selected and the number of cells in the membrane was counted.

### Wound healing assay

For the wound healing assay, control cells and different groups of gallbladder cancer cells were separately plated in 6-well plates. After complete attachment, a sterile tip was used to draw straight lines on the bottom of the plate. The plates were then washed with PBS for 3 times. The initial wound size was recorded using a microscope. After incubation at 37 °C for 24 h, the wound size was recorded.

### Plasmid construction

pcDNA3.1 plasmids encoding wide eEF1A2 and EEF1AKMT4 were purchased from Biosun Company. eEF1A2 K36R and EEF1AKMT4 D88A mutations were constructed through PCR-mediated site-directed mutagenesis using a QuickChange Site-Directed Mutagenesis Kit (Agilent Stratagene, CA, USA). The plasmids and primer sequences used were as follows.

**Table:** 

Plasmid	Vehicle information	Tag information
eEF1A2 WT	pcDNA3.1	FLAG
eEF1A2 K36R	pcDNA3.1	FLAG
EEF1AKMT4 WT	pcDNA3.1	–
EEF1AKMT4 D88A	pcDNA3.1	–

### Co-immunoprecipitation

The protein sample preparation was the same as that used for Western blotting before the addition of the loading buffer. To purify the eEF1A2 protein, every 1 mg protein sample was incubated with 2 μg primary antibody at 4 °C overnight. Protein A/G beads (MCE)were added and incubated at 4 °C for 2 h. Then, sediments were collected after centrifugation at 14,000 rpm at 4 °C for 1 min, washed three times with RIPA lysis buffer, then eluting with 40 μL 1x loading buffer at 95 °C for 5 min and used for Western blotting detection.

### Immunoprecipitation

For immunoprecipitation of endogenous eEF1A2, equal amounts of whole cell extracts (WCEs) were incubated with anti-eEF1A2 at 4 °C for overnight and then with protein magnetic beads at 4 °C for 2 h. The beads were washed with cell lysis buffer at 4 °C three times, boiled in Laemmli buffer, and then frozen until processed for MS analysis, as described below.

### Mass spectrometry of eEF1A2K36 methylation

Recombinant and immunoprecipitated eEF1A2 were separated by SDS-PAGE and stained with Coomassie blue. Bands were cut, and LC–MS/MS was authorized by Peking University (Thermo Scientific). All raw files were analyzed using the ByonicTM (Version 4.3.4) and ByologicTM software (Version 4.3.4; Protein Metrics Inc.) with a threshold control of 1% false discovery rate at protein level. The maximum mass deviations of the parent and fragment ions were set to 10 and 20 ppm, respectively. Carbamidomethyl was set as the fixed modification, while the oxidation and PTM of methyl (+14.015650 Da), dimethyl (+28.031300 Da), and trimethyl (+42.046950 Da) on lys residues were set as variable modifications. The methylation status of eEF1A2 K36 was inspected manually. Selected ion chromatograms for peptides spanning eEF1A2 K36 were extracted using Xcalibur Qual Browser (Thermo).

### Dot blotting assay

A suitable size of PVDF membrane (about 6 cm∗8 cm) was clipped and activated in methanol solution for 1 min. The membrane was then rinsed with ddH_2_O for three times and then dried at room temperature. The different types of modified peptides were diluted with PBS and then dropped onto PVDF membrane, and subsequently dried at 37 °C for about 45 min. After blocking with 5% skim milk powder and incubation with primary antibody and secondary antibody, dots were visualized using enhanced chemiluminescence (Millipore) according to the manufacturer's instructions.

### Purification of recombinant proteins

For purification of FLAG-tagged eEF1A2, eEF1A2 bearing a C-terminal Flag tag was expressed by transient transfection in wild-type or EEF1AKMT4-knocked HEK293T cells that were reconstituted with EEF1AKMT4 wild type (WT) or sh EEF1AKMT4, respectively. After 48 h of transfection, eEF1A2 was isolated from whole cell extracts using an anti-Flag M2 affinity gel (Sigma) according to the manufacturer's instructions and eluted with 3xFlag peptides (Sigma). Purified eEF1A2 was immediately used for the enzymatic reactions.

### GTPase assays

GTPase assays were performed using the ATPase/GTPase Activity Assay Kit (Abcam), following the manufacturer's protocol. Briefly, 3 μg of Flag-tagged eEF1A2K36me0/3 purified from HEK293T cells (as described above) was incubated with increasing amounts of GTP in 30 μL of the reaction buffer provided by the kit at 37 °C for 3 h. The reactions were terminated by adding 200 mL of the kit reagent and incubating for 30 min at room temperature. The formation of hydrolyzed free phosphate was measured at 620 nm, and the absorbance was compared with a standard curve. Readings of the background blank and negative control reactions were subtracted from the sample readings. The kinetic parameters were evaluated by fitting the data to the Michaelis–Menten equation in Graphpad prism 8.

### Translation assays

For SUnSET assays, wild type or shEEF1AKMT4 GBCSD and SGC996 cells were seeded at 2–4x10^5^ cells/mL in 6-well plates 24 h prior to serum starvation. For serum stimulation, cells were maintained in regular media containing 10% fetal bovine serum for an optimized period (2 h for GBCSD and 3 h for SGC996). Puromycin pulses were performed by incubating cells with 10 μg/mL puromycin for 15 min at 37 °C. Cells were then washed with cold PBS and lysed in RIPA buffer supplemented with 1 mM PMSF and a protease inhibitor mixture. A total of 5–10 mg of the whole cell lysate were assayed by Western blot analysis using an anti-puromycin antibody.

### *In vivo* xenograft studies

The experiment has passed the ethical review of experimental animal welfare by Shandong University Qilu Hospital. Approval number: Dwll-2024-358. Nude mice (BALB/c, female, 4–5 weeks of age, 14–16 g) were purchased from GemPharmatech Co., Ltd. (Nanjing, China) and randomly divided into groups (*n* = 6 per group). According to different groups, different types of cells were injected subcutaneously into the right flank to establish a xenograft model (approximately 1 × 10^6^ per mouse). Tumor diameter was measured every 3 days using a caliper. Three to four weeks after implantation, mice were sacrificed. The final tumor volume (V) was calculated using the following formula: V = (L × W^2^)/2, where L = length (mm) and W = width (mm).

Popliteal lymphatic metastasis model**.** BALB/c nude mice (4–5 weeks old, 14–16 g) were purchased from GemPharmatech Co., Ltd. (Nanjing, China) and randomly divided into groups (*n* = 6 per group). The footpads of mice were inoculated with 50 μL PBS suspensions of gallbladder cancer cells transduced with eEF1A2 WT, eEF1A2K36R, or shEEF1AKMT4. Four weeks after the injection, the mice were sacrificed. The size of the metastatic lymph nodes in the popliteal fossa was compared between the different groups. Popliteal lymph nodes were fixed with formaldehyde and embedded in paraffin. Then, HE staining confirmed the presence of popliteal lymph nodes.

### Statistical analysis

Statistical analysis was performed using SPSS 25.0 for Windows (SPSS Inc., Chicago, IL, USA). Continuous variables are expressed as mean ± standard deviation (SD) or median (interquartile range). Categorical variables were analyzed using the *χ*^2^ test or Fisher's exact test, and continuous variables were analyzed using the student's *t*-test. Univariate survival analysis was performed using the Kaplan–Meier (K–M) method. The relative prognostic significance of the variables for overall survival (OS) was evaluated using Cox proportional hazard regression models. All statistical tests were two-tailed, and *p* < 0.05 was considered significant. GraphPad Prism 8 (GraphPad Software, San Diego, CA, USA) was used for statistical analysis.

## Results

### eEF1A2 is tightly correlated with lymph node metastasis and poor prognosis in patients with GBC

**Figure 1:**
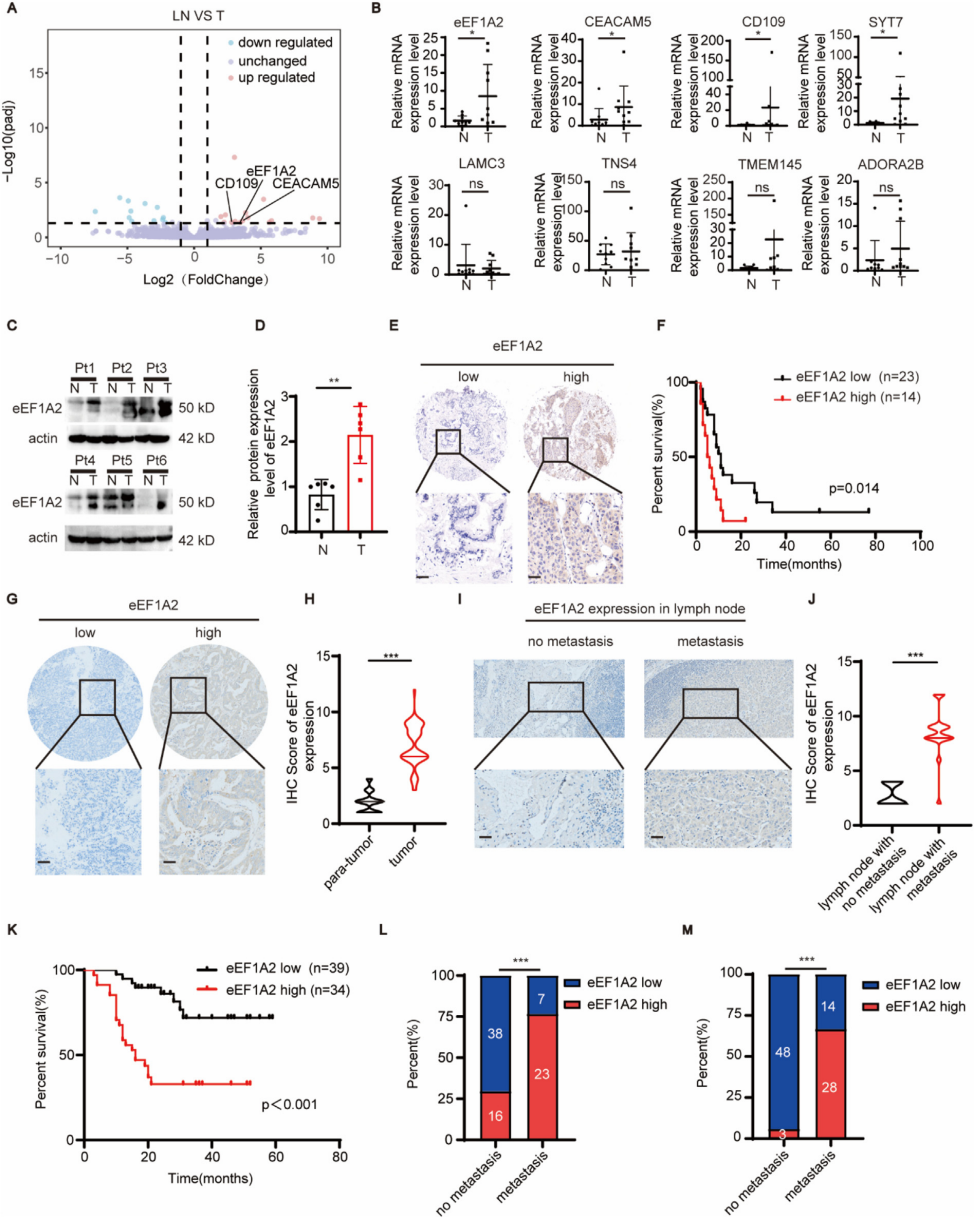
eEF1A2 is tightly correlated with lymph node metastasis and poor prognosis in patients with GBC. **(A)** Volcano plot showing DEGs of six pairs of tumors (T) and the corresponding metastasized lymph node tissues (LN). ∣log2FC∣ > 1, adjusted *p* value (*padj*) < 0.05. **(B)** Relative mRNA expression of 8 differentially expressed genes was further quantified by qRT-PCR in 10 paired gallbladder cancer tissues and adjacent normal tissues. **(C–D)** Expression of eEF1A2 in GBC tumor tissues and paired normal tissues was examined by Western blotting (C) and analyzed with ImageJ (D). **(E–F)** IHC staining was performed in a GBC microarray (cohort 1) to examine the expression of eEF1A2 in GBC tissues and its prognostic value. (E)Representative images of low and high eEF1A2 expression in Cohort 1. Scale bar: 100 μm. (F) Overall survival curves based on tumor eEF1A2 expression using the Kaplan–Meier method and analyzed by log-rank test. **(G)** Representative images of IHC staining for high and low eEF1A2 expression in GBC cohort 2. Scale bar: 100 μm. **(H)** IHC scores of eEF1A2 in GBC tumors and adjacent normal tissues in cohort 2 were analyzed. **(I)** Representative images of IHC staining for eEF1A2 expression in lymph nodes with no metastasis and those with GBC metastasis. Scale bar: 100 μm. **(J)** IHC scores of eEF1A2 expression in GBC lymph nodes with and without metastasis in cohort 2 were analyzed. **(K)** The overall survival curves based on tumor eEF1A2 expression in Cohort 2 were drawn using the Kaplan–Meier method and analyzed using the log-rank test. **(L–M)** Correlation analysis of tumor eEF1A2 expression and lymph node metastasis in gallbladder cancer cohort 1 (L) and cohort 2 (M). Statistical significance between groups was assessed using the chi-square test. Statistical significance between groups was assessed using Student's *t*-test. ns, not significant; ∗, *p* < 0.05; ∗∗ represents *p* < 0.01; ∗∗∗ represents *p* < 0.001.

To explore the potential molecular mechanisms of gallbladder cancer lymph node metastasis, 6 matched pairs of gallbladder cancer and the corresponding metastatic lymph node tissues were collected for transcriptome sequencing. The results revealed that 22 genes were significantly elevated in metastatic lymph node tissues compared to those in primary tumor tissues (Fig. 1A; Table S1). Among these 22 genes, 11 genes (GAGE4, GAGE6, GAGE7, GAGE12I, GAGE12G, ISY1-RAB43, IGHV3-38, GPRASP3, KRTAP4-1, KRT6B, RIMS2) had expression levels of 0 in the sequencing results of at least one sample and therefore they were not included in further analysis. In addition, there are three genes (GASK1B-AS1, KCNJ5-AS1, SYTL5) that have not been functionally reported or studied in the past which were also not included in further analysis. The remaining 8 genes, including eEF1A2, CEACAM5, CD109, SYT7, LAMC3, TNS4, TMEM145, and ADORA2B, were selected for further verification by qRT-PCR in 10 pairs of tumor tissues from patients with gallbladder cancer and the corresponding adjacent normal tissues. eEF1A2, CD109, CEACAM5, and SYT7 levels were significantly elevated in GBC tumor tissues (Fig. 1B). The relative expression levels of CD109 and SYT7 in GBC tumor tissues were lower (Fig. S1), and the difference in the expression of eEF1A2 between cancer and adjacent tissues was greater than that of CEACAM5 (5.25-fold vs 3.08-fold). Therefore, we chose eEF1A2 for further study.

Western blot analysis was performed on total protein lysates extracted from cancerous and adjacent normal tissues of patients with gallbladder cancer. The results revealed that the expression of eEF1A2 in cancer tissues was significantly elevated compared to that in the adjacent normal tissues (Fig. 1C–D). The expression of eEF1A2 in a GBC TMA (cohort 1) was examined using immunohistochemistry to evaluate the prognostic role of eEF1A2. Representative immunohistochemical images of high and low eEF1A2 expression in the GBC microarray are shown in Figure 1E. Kaplan–Meier analysis revealed that high eEF1A2 expression was associated with poor prognosis in patients with gallbladder cancer (Fig. 1F). A retrospective GBC cohort (cohort 2) containing GBC tumor tissues, adjacent normal tissues, normal lymph node tissues, and metastatic lymph node tissues was collected at our center from January 2017 to December 2020 to validate the prognostic value of eEF1A2. The expression of eEF1A2 was divided into low expression and high expression according to their IHC scores (Fig. 1G). The expression of eEF1A2 was significantly higher than that in normal tissues (Fig. 1H). The expression of eEF1A2 in both normal and metastatic lymph nodes was also analyzed (Fig. 1I). Interestingly, the expression of eEF1A2 in metastatic lymph nodes was significantly higher than that in normal lymph nodes (Fig. 1J). Kaplan–Meier analysis of cohort 2 also revealed that eEF1A2 expression was positively correlated with poor patient prognosis, similar to the results of cohort 1 (Fig. 1K).

Associations between eEF1A2 expression and clinicopathological characteristics were further evaluated using the chi-square test in both cohorts. The results revealed a significant correlation between eEF1A2 expression and lymph node metastasis (Fig. 1L and M). Patients with high eEF1A2 expression were also correlated with advanced T stage and TNM stage in cohort 1 (Table 1) and with tumor differentiation, advanced T stage, and TNM stage in cohort 2 (Table 2). Univariate and multivariate analyses were performed to identify independent prognostic factors of GBC. High eEF1A2 expression, higher T stage, and higher M stage were confirmed as prognostic factors of GBC, whereas none were further confirmed as independent prognostic factors in cohort 1 (Table 3). High eEF1A2 expression, poor tumor differentiation, higher T stage, higher N stage, and higher TNM stage were confirmed as prognostic factors of GBC, and high eEF1A2 expression, higher N stage, and higher TNM were further confirmed as independent prognostic factors of GBC in cohort 2 (Table 4).

**Table 1:** Correlations between eEF1A2 expression and clinicopathological characteristics in GBC cohort 1.

Characteristics	Category	GBC		*p*
		eEF1A2 low(*n* = 70)	eEF1A2 high(*n* = 70)	
Age(years)	<60	19	19	0.604
	≥60	51	50	
	N/A	0	1	
Gender	Male	25	23	0.722
	Female	45	47	
	N/A	0	0	
Tumor size(cm)	<4	35	25	0.202
	≥4	33	41	
	N/A	2	4	
Differentiation	Well/Moderate	31	34	0.611
	Poor	39	36	
	N/A	0	0	
T Stage	T1/T2	35	21	**0.046**
	T3/T4	25	32	
	N/A	10	17	
N stage	N0	38	16	**<0.001**
	N1/N2	7	23	
	N/A	25	31	
M Stage	M0	64	59	0.332
	M1	6	10	
	N/A	0	1	
TNM stage	Ⅰ-Ⅱ	17	3	**0.001**
	Ⅲ-Ⅳ	22	39	
	N/A	31	28	
Vascular invasion	Negative	3	41	0.649
	Positive	10	4	
	NA	57	61	

eEF1A2 Eukaryotic Translation Elongation Factor 1 Alpha 2, GBC gallbladder cancer.

**Table 2:** Correlations between eEF1A2 expression and clinicopathological characteristics in GBC cohort 2.

Characteristics	Category	GBC		*p*
		eEF1A2 low(*n* = 51)	eEF1A2 high(*n* = 42)	
Age(years)	<60	13	14	0.407
	≥60	38	28	
Gender	Male	29	22	0.666
	Female	22	20	
Tumor size(cm)	<4	34	23	0.241
	≥4	17	19	
Differentiation	Well/Moderate	37	22	**0.044**
	Poor	14	20	
T Stage	T1/T2	35	20	**0.040**
	T3/T4	16	22	
N stage	N0	48	14	**<0.001**
	N1/N2	3	28	
M Stage	M0	51	42	–
	M1	0	0	
TNM stage	Ⅰ-Ⅱ	34	7	**<0.001**
	Ⅲ-Ⅳ	17	35	
Vascular invasion	Negative	35	23	0.170
	Positive	16	19	

eEF1A2: eukaryotic translation elongation factor 1 alpha 2, GBC: gallbladder cancer.

**Table 3:** The prognostic significance of eEF1A2 and clinicopathological characteristics in GBC cohort 1.

Characteristics	GBC			
	Univariate analysis		Multivariate analysis	
	HR	p^a^	HR	p^b^
Age(years)				
<60	REF			
≥60	1.284	0.521		
Gender				
Male	REF			
Female	1.984	0.076		
Tumor size				
<4 cm		REF		
≥4 cm		1.225	0.606	
Differentiation				
Well/Moderate	REF			
Poor	1.550	0.237		
T stage				
T1/T2	REF		REF	
T3/T4	2.167	**0.042**	1.824	0.168
N stage				
N0	REF			
N1/N2	1.015	0.951		
M stage				
M0	REF		REF	
M1	3.250	**0.024**	2.615	0.095
TNM stage				
Ⅰ-Ⅱ	REF			
Ⅲ-Ⅳ	1.263	0.365		0
Vascular invasion				
Negative	REF			
Positive	1.525	0.268		
eEF1A2				
Low	REF		REF	
High	2.426	**0.022**	1.430	0.446

eEF1A2: Eukaryotic Translation Elongation Factor 1 alpha 2, GBC: gallbladder cancer, HR: hazard ratio.

a Calculated by log-rank test.

b Calculated by Cox-regression Hazard model.

**Table 4:** The prognostic significance of eEF1A2 and clinicopathological characteristics in GBC cohort 2.

Characteristics	GBC			
	Univariate analysis		Multivariate analysis	
	HR	p^a^	HR	p^b^
Age(years)				
<60	REF			
≥60	1.112	0.791		
Gender				
Male	REF			
Female	1.042	0.910		
Tumor size				
<4 cm	REF		REF	
≥4 cm	1.874	0.088	1.203	0.751
Differentiation				
Well/Moderate	REF		REF	
Poor	2.299	**0.023**	1.960	0.083
T stage				
T1/T2	REF			
T3/T4	4.244	**<0.001**		
N stage				
N0	REF		REF	
N1/N2	2.089	**0.047**	3.068	**0.026**
TNM stage				
Ⅰ-Ⅱ	REF		REF	
Ⅲ-Ⅳ	6.366	**<0.001**	5.979	**0.024**
Vascular invasion				
Negative	REF			
Positive	1.592	0.205		
eEF1A2				
Low	REF		REF	
High	2.426	**0.022**	4.726	**0.001**

eEF1A2: Eukaryotic Translation Elongation Factor 1 alpha 2, GBC: gallbladder cancer, HR: hazard ratio.

a Calculated by log-rank test.

b Calculated by Cox-regression Hazard model.

### eEF1A2 promotes the proliferation, migration and invasion ability of GBC cells

**Figure 2:**
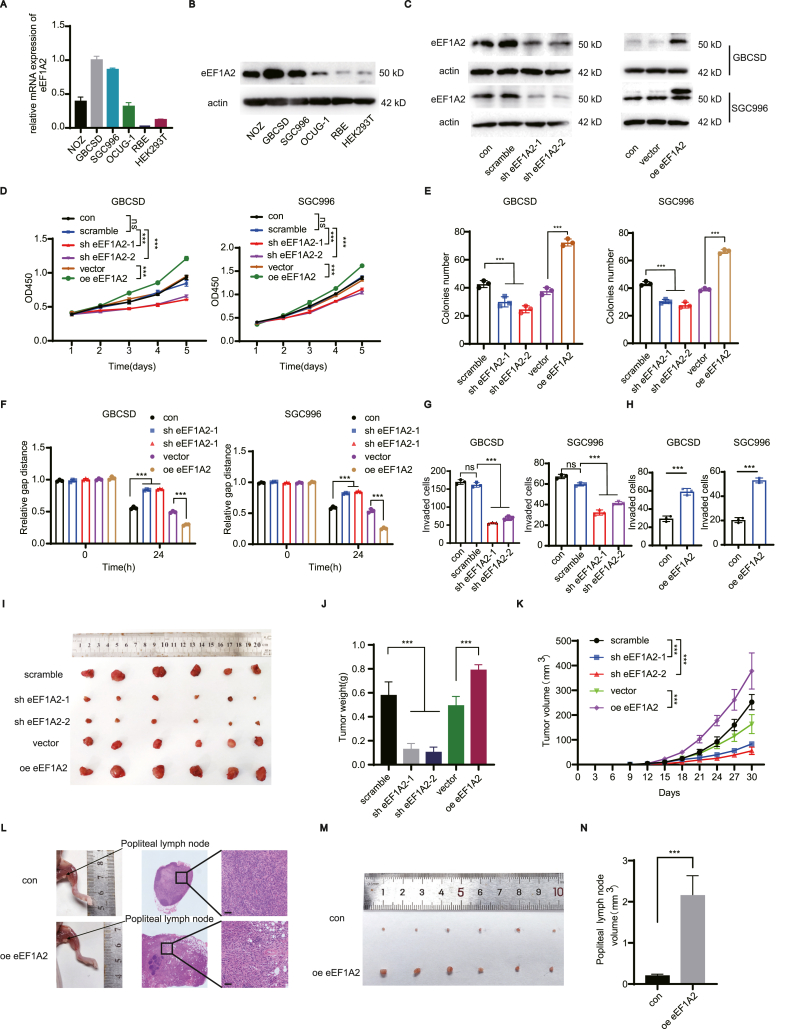
eEF1A2 exerts oncogenic effects and promotes GBC growth and metastasis. **(A, B)** Expression of eEF1A2 in the renal epithelial cell line HEK293T, cholangiocarcinoma cell line RBE and gallbladder cancer cell lines NOZ, GBCSD, SGC996, OCUG-1 were detected by qRT-PCR (A) and Western blot (B). **(C)** Western blot analysis of the knockdown and overexpression efficiency of eEF1A2 in the GBCSD and SGC996 cell lines. **(D)** Cell proliferation ability alterations after eEF1A2 knockdown and overexpression were detected with CCK8 assay in GBCSD and SGC996 cells. **(E)** Clone formation assay of cells with or without eEF1A2 knockdown and overexpression in GBCSD and SGC996 cells. **(F)** Wound healing assays were performed to investigate the effect of eEF1A2 knockdown and overexpression on the migration ability of the GBCSD and SGC996 cell lines. **(G–H)** Transwell assays with matrigel were performed to evaluate the invasive ability of GBCSD and SGC996 cells after eEF1A2 knockdown (G) and overexpression (H). **(I)***In vivo* subcutaneous xenografts were established using stable eEF1A2-overexpressing or eEF1A2-silenced GBCSD cells in nude mice, and the tumors were harvested 4 weeks later. Compared to the controls, knockdown of eEF1A2 significantly inhibited tumor growth, whereas overexpression of eEF1A2 significantly promoted tumor growth. **(J–K)** Tumor weight (J) and volume (K) of the subcutaneous xenografts were measured. **(L)** Representative images of a nude mouse model of popliteal LN metastasis. The indicated con and eEF1A2 overexpressing cells were injected into the footpads of nude mice. HE staining of the lymph nodes was performed to confirm the presence of metastasis.Scale bar: 100 μm. **(M)** Comparison of metastasized popliteal lymph node sizes in the control and eEF1A2 overexpression groups. **(N)** Comparison of the volume of metastasized popliteal lymph nodes in the control and eEF1A2 overexpression groups. Statistical significance between subgroups was assessed using the Student's t-test. ns, not significant; ∗∗∗, *p* < 0.001.

The intracellular function of eEF1A2 was then investigated in GBC cell lines. Expression of eEF1A2 in the renal epithelial cell line HEK293T, cholangiocarcinoma cell line RBE, and gallbladder cancer cell lines NOZ, GBCSD, SGC996, and OCUG-1 was examined by qPCR (Fig. 2A) and Western blotting (Fig. 2B). Based on the expression of eEF1A2 in cells, the GBCSD and SGC996 cell lines were selected for subsequent knockdown experiments. Stable eEF1A2 knockdown in GBCSD and SGC996 GBC cells was then established and validated via Western blotting (Fig. 2C) and qPCR (Fig. S2A). Although eEF1A2 showed high sequence similarity with eEF1A1, knockdown of eEF1A2 showed no effect on the mRNA expression of eEF1A1 (Fig. S2B). Stable eEF1A2 overexpression in all 4 GBC cell lines was then established and validated via Western blotting (Fig. 2C; Fig. S2D) and qPCR (Fig. S2C and S2D). CCK8 assays were performed to evaluate the effect of eEF1A2 knockdown or overexpression on the proliferation of GBC cell lines (Fig. 2D; Fig. S2E). Downregulation of eEF1A2 attenuated the proliferative ability of both GBCSD and SGC996 cell lines, and eEF1A2 overexpression extensively promoted GBC proliferation. Colony formation assays were performed to examine cell colony formation ability. Knockdown of eEF1A2 significantly weakened the colony formation ability of GBCSD and SGC996 cells, whereas eEF1A2 overexpression significantly enhanced the colony formation ability of GBC cells (Fig. 2E; Fig. S2F). Wound healing and transwell assays with matrigel were then performed to detect the effect of overexpression and knockdown of eEF1A2 on the ability of cell migration and invasion. The migration and invasion ability of gallbladder cancer cells was significantly weakened after eEF1A2 knockdown but enhanced after eEF1A2 overexpression (Fig. 2F–H; Fig. S2G–J).

### eEF1A2 promotes GBC tumor growth and lymph node metastasis *in vivo*

An *in vivo* experiment was performed to evaluate the proliferative function of eEF1A2 in GBC. First, stable cells of GBCSD with eEF1A2 knockdown or overexpression were injected subcutaneously into BALB/c nude mice to generate xenograft tumors. eEF1A2 knockdown substantially reduced the size of xenograft tumors, and the tumor size increased significantly after overexpression of eEF1A2 (Fig. 2I). Tumor weight and volume decreased significantly after eEF1A2 knockdown and increased significantly after eEF1A2 overexpression (Fig. 2J and K), indicating that eEF1A2 promotes tumor proliferation *in vivo*.

As eEF1A2 is significantly correlated with gallbladder cancer lymph node metastasis and eEF1A2 can promote the invasion and migration of gallbladder cancer cells, a foot pad popliteal lymph node metastasis model was established in nude mice to further evaluate the role of eEF1A2 in lymph node metastasis. Representative *in vivo* images of animals and HE staining results of popliteal lymph nodes are shown in Figure 2L. The lymph nodes were harvested 4weeks after inoculation of stable GBCSD cells overexpressing eEF1A2 or vector control into the foot pads of nude mice. Compared to the vector control group, overexpression of eEF1A2 significantly promoted the ability of GBCSD cells to metastasize to the popliteal lymph node (Fig. 2M). The tumor volume in the popliteal lymph nodes was significantly higher than that in the control group (Fig. 2N).

### EEF1AKMT4 trimethylates eEF1A2 at K36 site in GBC

**Figure 3:**
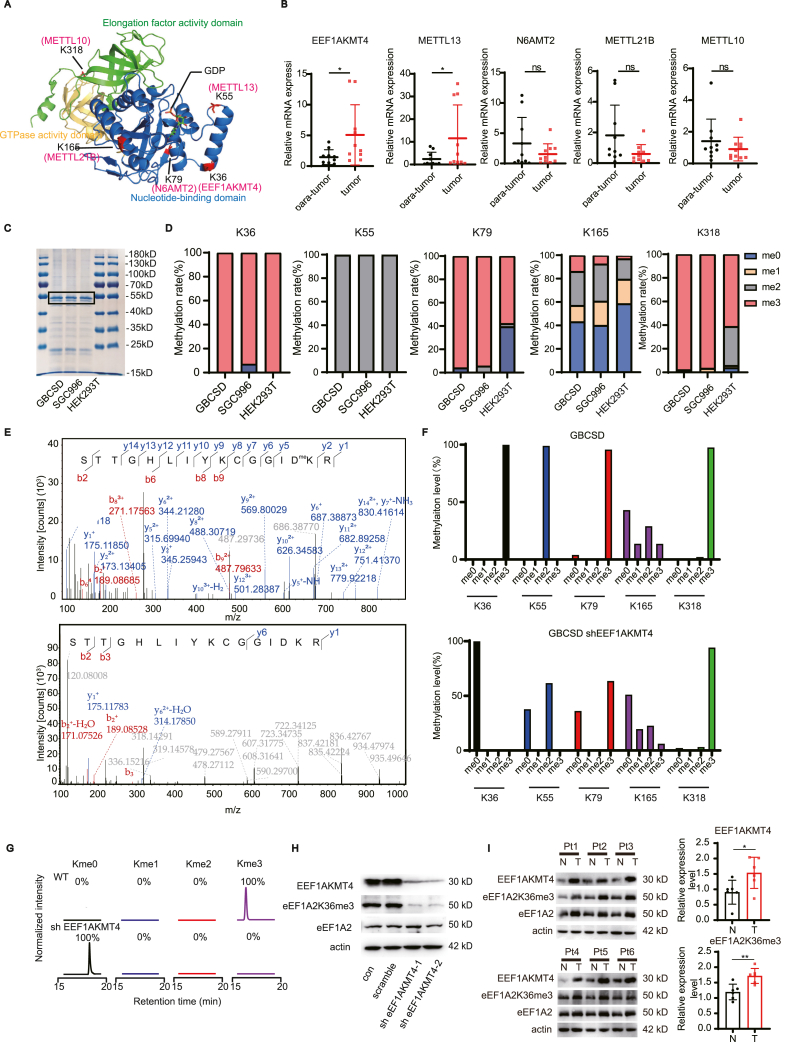
EEF1AKMT4 trimethylates eEF1A2 at K36 site in GBC. **(A)** Spatial distribution of the five main methylation sites in the protein structure of eEF1A2 combined with GDP (PDB: 6ra9). Most of the methylation sites were located in the nucleotide-binding domain of eEF1A2. **(B)** Relative mRNA expression of five methylases, EEF1AKMT4, METTL13, N6AMT2, METTL21B, and METTL10, which are responsible for methylation of K36, K55, K79, K165, and K318 sites, respectively, was quantified by qRT-PCR in 10 cases of gallbladder cancer tissues and their patient-paired normal tissues. **(C)** Coomassie blue staining of anti-eEF1A2 co-immunoprecipitation in GBCSD, SGC996, and HEK293T cell lines. Target bands are indicated by a black box and were cut and subjected to LC-MS/MS to analyze the methylation status. **(D)** Methylation status at the K36, K55, K79, K165, and K318 sites of eEF1A2 in GBCSD, SGC996, and HEK293T cell lines were analyzed. **(E)** Representative tandem mass spectra identifying *in vitro* tri-methylated (upper) and non-methylated (lower) eEF1A2K36. m/z for b and y ions observed in the spectra are indicated in red and blue, respectively. **(F)** Histogram showing the methylation changes in K36, K55, K79, K165, and K318 before (upper) and after (lower) knockdown of EEF1AKMT4 in GBCSD cells. **(G)** Selected ion chromatograms for non-, mono-, di-, and trimethyl eEF1AK36 peptides from GluC digestion of endogenous eEF1A2 immunoprecipitated from whole-cell lysates of GBCSD, indicating the methylation status shift after EEF1AKMT4 knockdown. **(H)** Western blot analysis of the knockdown efficiency of EEF1AKMT4 in GBCSD and the status of eEF1A2 and eEF1A2K36me3. **(I)** Expression of eEF1AKMT4 and eEF1A2 K36me3 levels in GBC tumor tissues and paired normal tissues were examined by Western blotting and analyzed using ImageJ. Statistical significance between groups was assessed using Student's t-test. ns, not significant; ∗, *p* < 0.05; ∗∗, *p* < 0.01.

eEF1A2 is a highly methylated molecule that is highly conserved between yeast and humans. The distribution of all five methylation sites was analyzed to determine the function of methylation of eEF1A2. As shown in Figure 3A, four of the five methylation sites were located at the nucleotide-binding domain, and importantly, the K36 and K55 sites were both located near the GDP-binding pocket, indicating a significant role of protein methylation in nucleotide-binding and enzyme activity. As methylation of each site is specifically carried out by a single methylase, the expression of five methylases was detected in 10 pairs of gallbladder cancer and adjacent tissues. The expression of EEF1AKMT4, which specifically catalyzes the methylation of K36 and METTL13, which specifically catalyzes the methylation of K55^24^ was significantly increased in tumor tissues compared to adjacent normal tissues (Fig. 3B). The methylation status of eEF1A2 in GBC was then evaluated. The eEF1A2 protein in three cell lines, GBCSD, SGC996, and HEK293T, was purified by anti-eEF1A2 co-immunoprecipitation and stained with Coomassie blue (Fig. 3C). The gel was then cut and subjected to liquid chromatography-tandem mass spectrometry (LC-MS/MS) to analyze the methylation status of eEF1A2. eEF1A2 was trimethylated in GBC cell lines at K36, K79, and K318, and dimethylated at K55 and K165 (Fig. 3D). Meanwhile, K36 and K55 were methylated nearly 100% in all three cell lines, whereas methylation levels of K79 and K318 differed among the three cell lines. In addition, some sites with relatively low methylation, such as K244 and K408, were also identified (data not shown).

We then examined the role of METTL13-regulated K55 methylation in promoting GBC LN metastasis by RNA interference of METTL13 in two GBC cell lines (Fig. S3A). Interestingly, downregulation of METTL13 significantly attenuated the proliferation, migration and invasion ability of GBCSD and SGC996 cell lines indicating a significant role of METTL13-regulated K55 methylation in promoting GBC LN metastasis (Fig. S3B–D). However, METTL13-regulated K55 methylation and subsequent enhancement of translational output is a well-known tumor promoting mechanism.^24^ And therefore, we then focused on the function and mechanism of K36 methylation in promoting GBC progression. The efficacy and specificity of EEF1AKMT4 as a methylase of eEF1A2 K36 has been evaluated by knockdown of EEF1AKMT4. Although eEF1A1 was also identified as a methylation substrate of EEF1AKMT4, there was no difference in the expression level between GBC tumor tissues and adjacent normal tissues indicating a less important role of eEF1A1 in the tumorigenesis and lymph node metastasis of GBC (Fig. S4). Representative mass spectrometry eEF1A2 K36 peptide and K36me3 peptide was shown in Figure 3E. Meanwhile, knockdown of EEF1AKMT4 significantly reduced the EEF1A2 K36 trimethylation level with only slightly affecting the methylation of K55, K79, K165, and K318 (Fig. 3F). Representative tandem mass spectra identifying *in vitro* tri-methylated and non-methylated eEF1A2 K36 were also shown in Figure 3G. An eEF1A2 K36 trimethylation detection antibody was then produced to further evaluate the effect of K36 trimethylation on eEF1A2 function. Dot blotting assay revealed a high efficacy and specificity of the eEF1A2K36me3 detection antibody (Fig. S5). As shown in Figure 3H, the Western blot results were consistent with the LC-MS/MS findings, further confirming the specificity and efficacy of the EEF1A2 K36me3 antibody. The expression of EEF1AKMT4 and methylation level of eEF1A2 K36 in tumor tissues and corresponding adjacent normal tissues were then examined. The results revealed that, compared with adjacent normal tissues, the expression of EEF1AKMT4 and the methylation degree of eEF1A2 K36 were significantly upregulated in GBC tumor tissues (Fig. 3I).

### Knockdown of EEF1AKMT4 inhibits the malignant phenotype of GBC while its overexpression is not tumor-promoting

**Figure 4:**
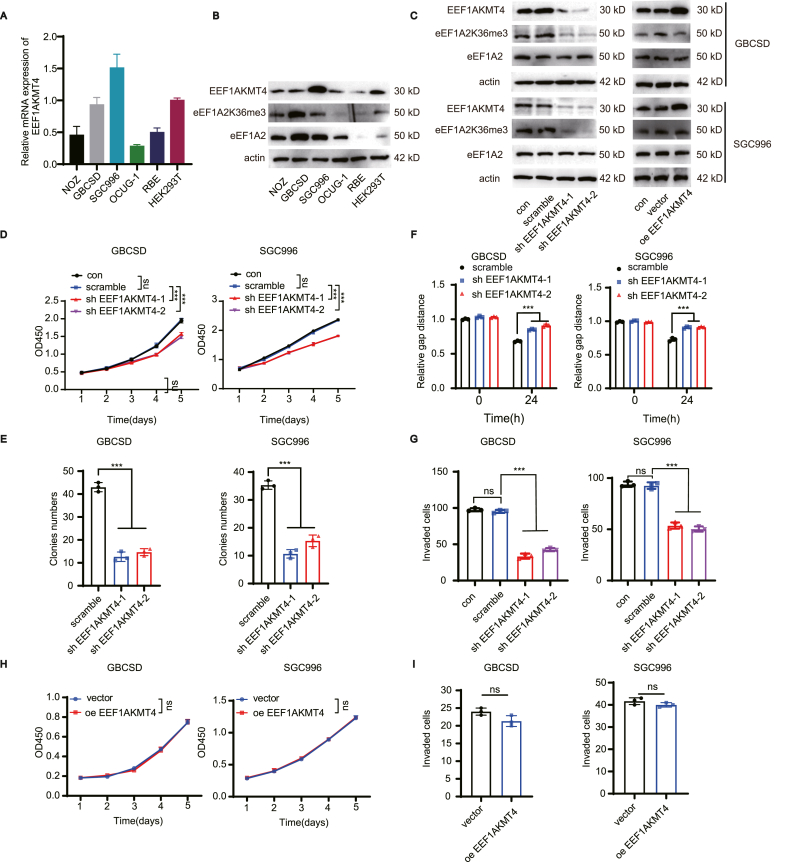
Knockdown of EEF1AKMT4 inhibits the malignant phenotype of GBC while its overexpression is not tumor-promoting. **(A–B)** Expression levels of EEF1AKMT4 in HEK293T, RBE, NOZ, GBCSD, SGC996, and OCUG-1 were detected using qRT-PCR (A) and Western blotting (B). **(C)** EEF1AKMT4 was knocked down or overexpressed in the GBCSD and SGC996 cell lines, and validated by Western blotting. **(D)** CCK-8 assays were performed to identify alterations in cell proliferation after EEF1AKMT4 knockdown in GBCSD and SGC996 cells. **(E)** Clone formation assay was performed in EEF1AKMT4 knockdown and vector control cells in GBCSD and SGC996 cells. **(F)** Wound healing assays were performed to investigate the effect of EEF1AKMT4 knockdown on the migration ability of the GBCSD and SGC996 cell lines. **(G)** Transwell assays with matrigel were performed to evaluate the invasive ability of GBCSD and SGC996 cells after EEF1AKMT4 knockdown. **(H)** CCK-8 assays were performed to identify alterations in cell proliferation after EEF1AKMT4 overexpression in GBCSD and SGC996 cells. **(I)** Transwell assays with matrigel were performed to evaluate the invasive ability of GBCSD and SGC996 cells after EEF1AKMT4 overexpression. Statistical significance between subgroups was assessed using the Student's t-test. ns, not significant; ∗∗∗, *p* < 0.001.

The intracellular function of EEF1AKMT4 in gallbladder cancer was also evaluated. The expression of EEF1AKMT4 in the HEK293T, RBE, NOZ, GBCSD, SGC996, and OCUG-1 was examined by qPCR (Fig. 4A) and Western blotting (Fig. 4B). Stable GBCSD and SGC996 GBC cells with EEF1AKMT4 knockdown and overexpression were then established and validated via Western blotting and qPCR (Fig. 4C; Fig. S6A, 6B), and the level of eEF1A2 K36me3 was also examined (Fig. 4C). CCK8 assays were performed to evaluate the effect of EEF1AKMT4 knockdown on the proliferation of GBCSD and SGC996 cells (Fig. 4D). Downregulation of EEF1AKMT4 attenuated the proliferation of both GBCSD and SGC996 cells. Colony formation assays revealed that the colony formation ability of EEF1AKMT4-silencing GBCSD and SGC996 cells was significantly weakened (Fig. 4E and Supplemental Fig. 6C). Wound healing and transwell assays with matrigel were then performed to detect the effect of EEF1AKMT4 on cell invasion and migration (Fig. 4F–G and Supplemental Fig. 6D and 6E). The invasion and migration of gallbladder cancer cells were significantly weakened after EEF1AKMT4 knockdown.

Interestingly, overexpression of EEF1AKMT4 showed no enhancement on the level of eEF1A2 K36me3 (Fig. 4C). Meanwhile, no significant changes in the proliferation and invasion abilities of GBC cell lines were observed (Fig. 4H–I and Supplemental Fig. 6F). Meanwhile, the expression of EEF1AKMT4 was also examined in metastatic lymph nodes and primary GBC tumors. The results revealed that there was no significant difference in the protein expression levels of EEF1AKMT4 between metastatic lymph nodes and primary GBC tumors (Supplemental Fig. 6G). Collectively, these results revealed that the effect of EEF1AKMT4 on the function of GBC cells depends on the methylation level of eEF1A2 K36.

### K36 site trimethylation is essential for the tumor-promoting effect of eEF1A2 *in vitro*

**Figure 5:**
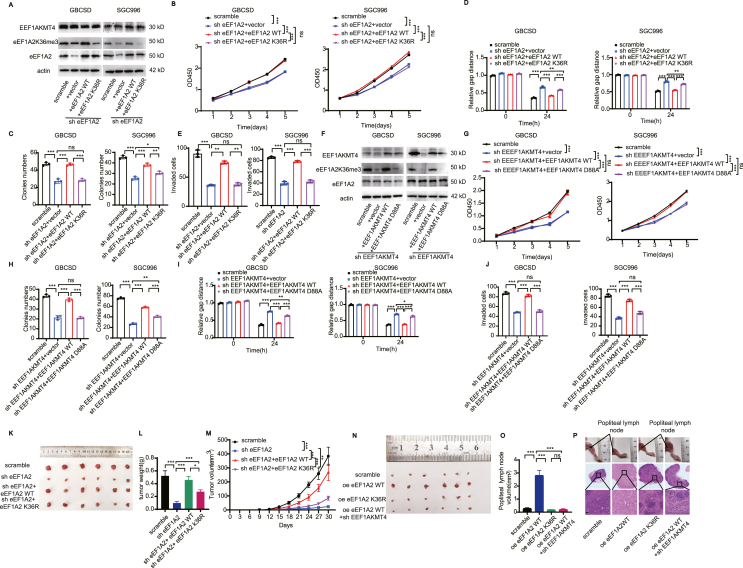
K36 site trimethylation is essential for the tumor-promoting effect of eEF1A2. **(A)**Western blot analysis of the expression of EEF1AKMT4, eEF1A2 K36me3, and eEF1A2 in eEF1A2-knockdown GBCSD SGC996 cells complemented with vector, eEF1A2 WT, or eEF1A2 K36R. **(B)** CCK-8 assays in eEF1A2-knockdown GBCSD SGC996 cells complemented with vector, eEF1A2 WT, or eEF1A2 K36R. **(C)** Clone formation assays of cells treated with scrambled shRNA, or eEF1A2-knockdowned GBCSD SGC996 cells complemented with vector, eEF1A2 WT or eEF1A2 K36R. **(D)** Wound healing assays of cells treated with scrambled shRNA, or eEF1A2-knockdowned GBCSD SGC996 cells complemented with vector, eEF1A2 WT or eEF1A2 K36R. **(E)** Transwell assays with matrigel of cells treated with scrambled shRNA, or eEF1A2-knockdowned GBCSD SGC996 cells complemented with the vector, eEF1A2 WT, or eEF1A2 K36R. **(F)** Western blot analysis of the expression of EEF1AKMT4, eEF1A2 K36me3, and eEF1A2 in EEF1AKMT4-knockdown GBCSD cells complemented with vector, EEF1AKMT4 WT, or EEF1AKMT4 D88A. **(G)** CCK-8 assays in EEF1AKMT4-knockdown GBCSD SGC996 cells complemented with vector, EEF1AKMT4 WT or EEF1AKMT4 D88A. **(H)** Clone formation assays of cells treated with scrambled shRNA, or EEF1AKMT4-knockdowned GBCSD SGC996 cells complemented with vector, EEF1AKMT4 WT or EEF1AKMT4 D88A. **(I)** Wound healing assays of cells treated with scrambled shRNA, or EEF1AKMT4-knockdowned GBCSD SGC996 cells complemented with vector, EEF1AKMT4 WT or EEF1AKMT4 D88A. **(J)** Transwell assays with matrigel of cells treated with scrambled shRNA, or EEF1AKMT4-knockdowned GBCSD SGC996 cells complemented with the vector, EEF1AKMT4 WT, or EEF1AKMT4 D88A. **(K)***In vivo* subcutaneous xenografts were established. **(L–M)** Tumor weight (L) and volume (M) of the subcutaneous xenografts were measured. **(N)** Comparison of popliteal lymph node size. **(O)** Comparison of the volume of metastasized popliteal lymph nodes. **(P)** Representative images of a nude mouse model of popliteal LN metastasis. HE staining of the lymph nodes was performed. Scale bar: 100 μm ns, not significant; ∗*p* < 0.05; ∗∗*p* < 0.01; ∗∗∗*p* < 0.001.

Experiments were then conducted to verify the effect of K36 site trimethylation on the intracellular function of GBC cells. Lysine (K) to arginine (R) were usually applied to abolish the modification of lysine without changing the electric charge and protein physicochemical property. The effect of K36 site trimethylation on the intracellular function of GBC cells were then analyzed using the eEF1A2 K36R mutation. The methylation changes at the K36 site in GBCSD and SGC996 cells after eEF1A2 knockdown complemented with vector, eEF1A2 WT or eEF1A2 K36R were examined by Western blotting (Fig. 5A). CCK8 assays were performed to evaluate the effect of K36R mutation on cell proliferation (Fig. 5B). Supplement of K36R mutation mimics the effect of eEF1A2 knockdown on cell proliferation. Colony formation assays, wound healing and transwell with matrigel assays were also performed to detect the effects of K36R mutation on cell clone formation, migration and invasion abilities (Fig. 5C–E and Supplemental Fig. 7A–7C). The results revealed that compared to eEF1A2 knockdown, the proliferation, migration, and invasion abilities of GBC cells were recovered after EEF1AKMT4 WT supplementation, but there was no significant effect after eEF1A2 K36R supplementation.

As D88A mutation eliminated the catalytic function of EEF1AKMT4 and EEF1AKMT4 is a specific methylase of eEF1A2 K36, further experiments were also conducted to verify the effect of K36me3 on the intracellular function of GBC cells by knocking down of EEF1AKMT4 and introducing of EEF1AKMT4 D88A mutation. The methylation changes at the K36 site in GBCSD and SGC996 cells after EEF1AKMT4-knockdowned complemented with vector, EEF1AKMT4 WT or EEF1AKMT4 D88A were examined by Western blotting (Fig. 5F). Knockdown of EEF1AKMT4 significantly reduced the eEF1A2K36me3 level while supplement of EEF1AKMT4 D88A didn't reverse this reduction. CCK8 assays were then performed to evaluate the effect of EEF1AKMT4 D88A on cell proliferation (Fig. 5G). Colony formation assays, wound healing and transwell with matrigel assays were performed to detect their effects on cell clone formation, migration and invasion ability (Fig. 5H–J and Supplemental Fig. 7D–7F). Compared to EEF1AKMT4 knockdown, the proliferation, migration, and invasion abilities of gallbladder cancer cells were enhanced after EEF1AKMT4 WT supplementation, but there was no significant effect after EEF1AKMT4 D88A supplementation indicating that eEF1A2K36me3 has no tumor promoting effect. Overall, these results revealed that K36 site trimethylation is essential for the tumor-promoting effect of eEF1A2.

### K36 site trimethylation is essential for eEF1A2 induced GBC tumor growth and lymph node metastasis *in vivo*

The function of EEF1AKMT4-eEF1A2K36me3 in GBC was then verified *in vivo*. First, the stable cells of GBCSD with eEF1A2 knockdown, eEF1A2 knockdown complemented with eEF1A2WT, or eEF1A2K36R were injected subcutaneously into BALB/c nude mice to generate xenograft tumors. eEF1A2 knockdown significantly reduced the size of xenograft tumors, whereas supplementation with eEF1A2 WT significantly increased tumor size. Supplementation with eEF1A2K36R showed no significant change in tumor size compared to that in the eEF1A2 knockout group (Fig. 5K). Tumor weight and volume decreased significantly after eEF1A2 knockdown and increased significantly after supplementation with eEF1A2 WT. However, the weight and volume did not significantly change after supplementation with eEF1A2K36R (Fig. 5L–M). These results indicate that eEF1A2K36me3 promotes tumor proliferation *in vivo*.

A footpad lymph node metastasis model in nude mice was also conducted to further evaluate the role of eEF1A2K36me3 in lymph node metastasis. Stable GBCSD cells overexpressing eEF1A2, GBCSD cells overexpressing eEF1A2K36R, and GBCSD cells overexpressing eEF1A2 WT with EEF1AKMT4 knockdown were inoculated into the foot pads of nude mice, and the metastatic lymph nodes were harvested after 4 weeks. Compared with the control group, overexpression of eEF1A2 significantly promoted GBC lymph node metastasis, whereas overexpression of eEF1A2K36R or knockdown of EEF1AKMT4 after overexpression of eEF1A2 had no significant effect on lymph node metastasis (Fig. 5N). The volume of popliteal lymph nodes in the eEF1A2 overexpression group was significantly higher than that in the control group; however, there was no significant change in the volume of popliteal lymph nodes after eEF1A2K36R overexpression and EEF1AKMT4 knockdown after eEF1A2 overexpression (Fig. 5O). Representative *in vivo* images of animals and HE staining results of popliteal lymph nodes are shown in Fig. 5P. These results revealed a main role of the trimethylation of eEF1A2 K36me3 in the progression and lymph node metastasis of GBC.

### eEF1A2 K36 trimethylation affects protein output by affecting its GTP enzyme activity

**Figure 6:**
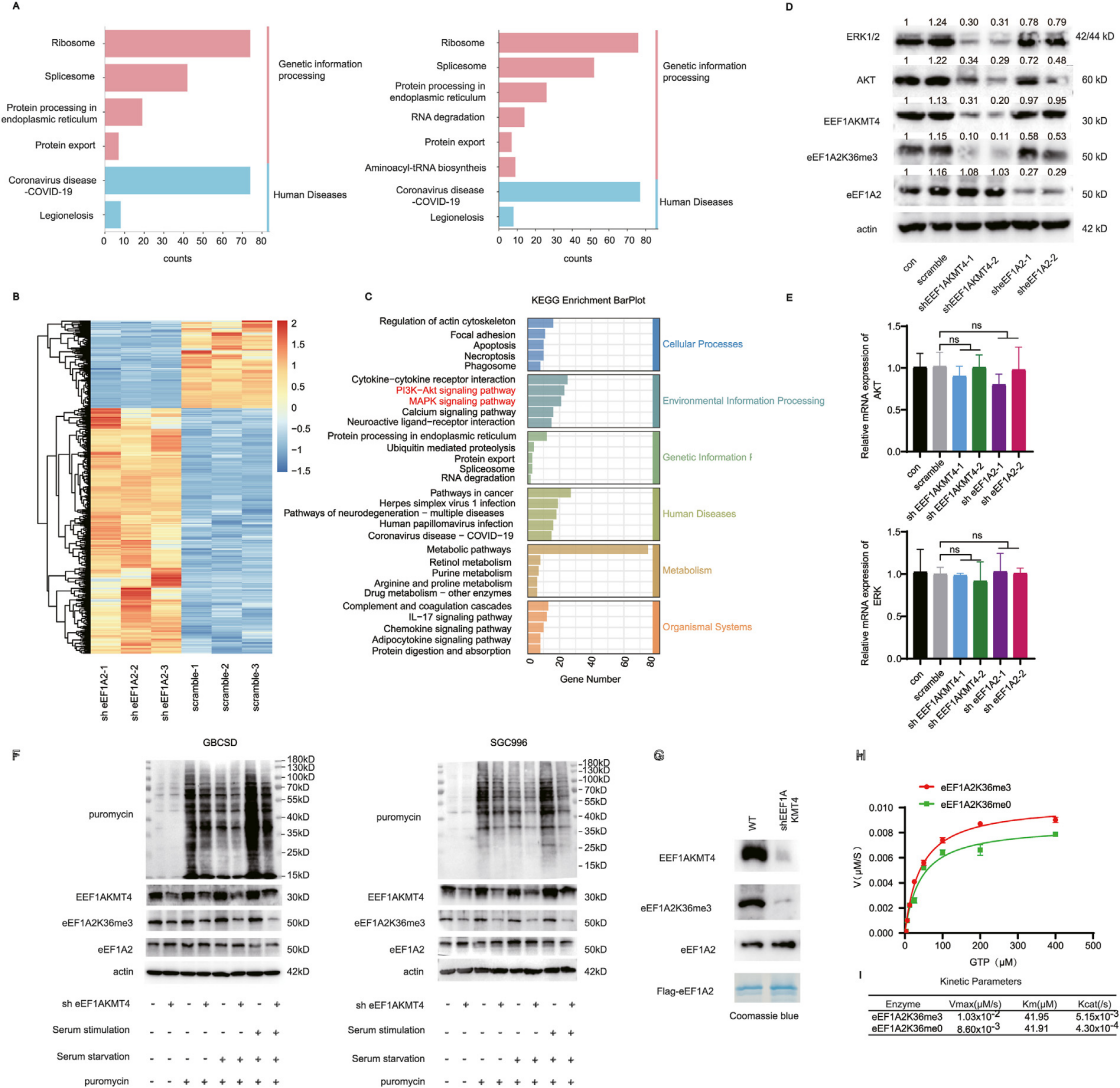
eEF1A2 K36 trimethylation affects protein output by affecting its GTP enzyme activity. **(A)** FLAG-tagged eEF1A2 WT and eEF1A2 K36R were overexpressed in HEK293T cells. Flag-co-IP and subsequent LC-MS/MS were performed to identify the differences in binding proteins between eEF1A2 WT and K36R mutations. The binding proteins of eEF1A2 WT (left) and eEF1A2 K36R (right) in HEK293T cells were analyzed by KEGG enrichment analysis. **(B)** Heatmap showing the differentially expressed genes after eEF1A2 knockdown in GBCSD cell lines. **(C)**The differentially expressed genes after eEF1A2 knockdown were analyzed by KEGG enrichment analysis. **(D)** Western blot analysis of the expression of ERK1/2 and AKT in EEF1AKMT4-knockdown or eEF1A2-knockdown GBCSD cells. **(E)** qPCR analysis of the mRNA expression of ERK1/2 and AKT in EEF1AKMT4-knockdown or eEF1A2-knockdown GBCSD cells. **(F)** SUnSET assays were performed under the indicated conditions to analyze the effect of eEF1A2 K36 methylation status on the protein synthesis rate in GBCSD (left) and SGC996(right) cell lines. These results revealed reduced protein production in eEF1A2 K36me0 cells. **(G)** Purification of eEF1A2 ± K36me3 protein with anti-eEF1A2 co-immunoprecipitation in EEF1AKMT wt and knockdown cells. Top panel: Western blot validation of EEF1AKMT4; middle panels: Western blot analysis with the indicated antibodies against eEF1A2 purified from 293 T cells; bottom panel: Coomassie stain of purified eEF1A2 protein. **(H)***In vitro* GTP hydrolysis by trimethylated or unmethylated eEF1A2. eEF1A2 ± K36me3 purified was incubated with increasing amounts of GTP at 37 °C for 3 h. Kinetic parameters were obtained by fitting the Michaelis–Menten equation to plot the velocity of phosphate formation against GTP concentration. **(I)** K36me3 increases the catalytic efficiency of GTP hydrolysis by eEF1A2. The Michaelis–Menten kinetic parameters of eEF1A2 ± K36me3 are shown.

To further analyzed the potential mechanism underlying eEF1A3K36me3 induced tumor progression, co-IP and mass spectrometry were then performed to determine whether there was any change in the binding proteins after mutation of the eEF1A2 K36 site. KEGG enrichment analysis of the proteins identified by mass spectrometry showed that there were only slightly differences between the binding proteins of eEF1A2 WT and K36R (Fig. 6A). The differentially expressed genes (DEGs) after eEF1A2 knockdown in GBCSD cell lines were also analyzed with transcriptome sequencing (Fig. 6B). KEGG enrichment analysis revealed that the DEGs enriched in the PI3K-AKT signaling and MAPK signaling pathway (Fig. 6C). The protein expression of ERK1/2 and AKT after knockdown of eEF1A2 or EEF1AKMT4 was then analyzed. Knocking down of eEF1A2 attenuated the expression level of total eEF1A2 and therefore affecting the level of eEF1A2K36me3, resulting in reduced expression of ERK1/2 and AKT. Meanwhile, knocking down of EEF1AKMT4 resulted in nearly abolish of the level of eEF1A2K36me3 and a more pronounced downregulation of ERK1/2 and AKT expression levels (Fig. 6D). These results indicate that the level of eEF1A2K36me3 instead of the expression of eEF1A2 is a more prominent effector of ERK1/2 and AKT expression. However, the mRNA expression of ERK1/2 and AKT has not been affected knockdown of eEF1A2 or EEF1AKMT4 (Fig. 6E), indicating that eEF1A2K36me3 may affects the expression of ERK1/2 and AKT by affecting the translation efficiency.

**Figure 7:**
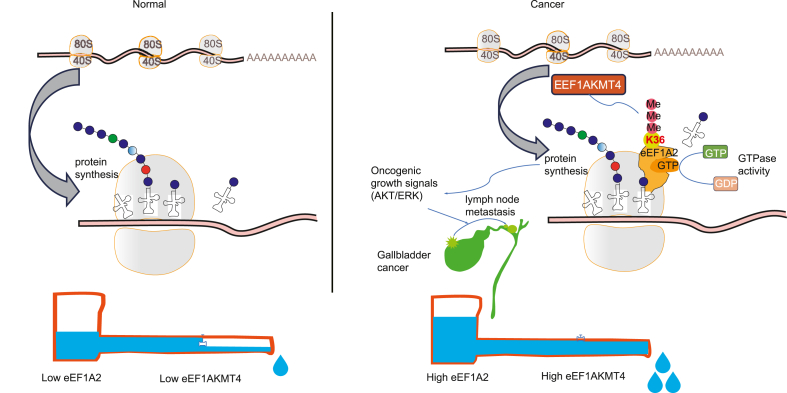
A schematic model shows the effects of EEF1AKMT4 and eEF1A2 on regulating ribosomal protein synthesis. EEF1AKMT4 trimethylates eEF1A2 at K36 and fuels its GTPase activity. Elevated eEF1A2 enzyme activity promotes the protein output of several oncogenic growth signals including AKT and ERK. Overall, upregulated eEF1A2 expression together with the fuel of EEF1AKMT4 promotes the progression of GBC and LN metastasis.

Since K36 of eEF1A2 is located on the catalytic surface of the eEF1A2 nucleotide-binding domain and near the GDP binding pocket (Fig. 3A), we hypothesized that methylation of the K36 site may affect the GTPase activity of eEF1A2, thus affecting mRNA translation. Therefore, surface sensing of translation (SUnSET) was performed to detect changes in total protein synthesis in GBCSD and SGC996 cells (Fig. 6F). In both cell types, total protein synthesis was reduced after EEF1AKMT4 knockdown, and this effect was more prominent when the serum was re-administered after starvation. Next, FLAG-tagged eEF1A2 from EEF1AKMT4 wild-type (WT) or almost completely knocked down HEK293T cells was further purified (Fig. 6G). *In vitro* GTP hydrolysis experiments were performed to determine the Michaelis–Menten kinetic properties of purified eEF1A2 ± K36me3 (Fig. 6H). Compared with K36me0 eEF1A2, the *V*_*max*_ increased after K36 trimethylation and, *K*_*m*_ did not change significantly, and the catalytic efficiency of eEF1A2 increased by approximately 20% (Fig. 6I). Therefore, EEF1AKMT4-dependent eEF1A2 K36 trimethylation increased the GTPase activity of eEF1A2 and enhanced tumor promoting signals including ERK1/2 and AKT by promoting the total protein synthesis in GBC cells (Fig. 7).

## Discussion

Gallbladder cancer is the most common tumor of the biliary system and has poor prognosis. Treatment selection for GBC is limited, with surgery being the only possible cure. Lymph node metastasis is correlated with an advanced stage of disease and is also a key factor affecting the overall outcome after radical surgery in T2 stage GBC.^25^ The discovery of the molecular mechanisms related to lymph node metastasis of gallbladder cancer and the identification of new promising therapeutic sites are urgently needed for the prevention and treatment of gallbladder cancer. Studies on the unique mechanism of tumor remodeling, tumor evolution, immune re-education, and tumor-mesenchymal cell crosstalk during lymph node metastasis of GBC are still lacking. Previous studies by our team have shown that elevated bile acids (BA) can increase the transcription and expression of fibroblast growth factor 19 (FGF19) and fibroblast growth factor receptor (FGFR4) by activating the GPBAR1-cAMP-EGR1 pathway. FGF19 promotes lymph node metastasis and progression of GBC via autocrine stimulation of FGFR4 and downstream ERK through bile.^26^ In this study, we collected six pairs of matched GBC cancers and pathologically proven metastatic lymph nodes for transcriptomic sequencing and found that eEF1A2 expression was upregulated in gallbladder cancer. We also found that high eEF1A2 expression was closely associated with lymph node metastasis in the two GBC cohorts. As one of the key participants in ribosome mRNA translation, the findings of the significant role of eEF1A2 in GBC lymph node metastasis, together with the recent findings of other groups,^24^^,^^27^ have confirmed the important but unrevealed role of translation efficiency and thus protein synthesis in malignant tumor progression and metastasis.

eEF1A2 is a member of the eEF1A gene family that functions as a transporter that brings amino acids into the polypeptide chain and matches the codon on mRNA to facilitate protein synthesis.^28^ Mutations in eEF1A2 are associated with neurological diseases such as neurodegenerative diseases.^29^ Studies have also shown that eEF1A2 is an oncogene that promotes tumor progression in ovarian, lung, and pancreatic cancers.^10^^,^^11^^,^^30^ In our study, we found that eEF1A2 knockdown impaired cell proliferation, migration, and invasion *in vitro* and inhibited tumor growth and lymph node metastasis *in vivo*, whereas overexpression of eEF1A2 promoted these processes in two GBC cell lines. As eEF1A2 affects the function of GBC cells in multiple dimensions, we hypothesized a broader effect of eEF1A2 in cell biology, instead of combining it with a particular protein.

Non-histone methylation refers to the modification of methylation of non-histone proteins that do not involve chromatin structure in cells.^31^ Non-histone methylation is an important form of protein modification that plays an important regulatory role in various biological processes.^32^ Our results revealed that eEF1A2 was highly methylated in GBC cells. The methylation sites of eEF1A2 in GBC cells include but are not limited to K36, K55, K79, K165, K318, and others. The K36 and K55 sites were hypermethylated in all three examined cell lines, including two GBC cell lines and HEK293T cell line. Meanwhile, the methylases of K36 and K55, namely EEF1AKMT4 and METTL13,^17^^,^^24^ were also highly expressed in GBC tissues compared with adjacent normal tissues. Since the function of K55 methylation in tumors has been elucidated,^24^ we selected the K36 site for further investigation. Knockdown of EEF1AKMT4 significantly affected the proliferation, migration, and invasion abilities of gallbladder carcinoma cells; however, there was no significant change in cell function after overexpression of EEF1AKMT4, suggesting that the methylation status of eEF1A2 K36 plays an important role in the function of gallbladder cancer cells.

eEF1A2 consists of three main functional domains: the nucleotide binding domain, elongation factor activity domain, and GTPase activity domain.^33^ Different domains of eEF1A2 cooperate with each other to ensure efficient protein translation and synthesis. The GTPase active domain is responsible for hydrolyzing GTP to GDP and releasing the corresponding energy, a process critical for regulating the rate of protein synthesis. The nucleotide-binding domain works in concert with the GTPase activity domain to regulate different stages of protein synthesis through the binding of GTP or GDP. Tumor cells have greater energy expenditure and protein output than normal cells.^34^ K36 is located on the nucleotide-binding domain; therefore, we hypothesized that methylation of K36 may affect the GTPase activity of eEF1A2. Through kinetic analysis and the SUnSET assay,^35^ we demonstrated that K36me3 enhanced the GTPase activity of eEF1A2 and promoted the translation process and protein synthesis. These results revealed the significant role of protein methylation in affecting its function. We also demonstrated that the trimethylation of eEF1A2 K36 is a crucial part of protein synthesis, controlling the rate and accuracy of protein synthesis.

As an important intracellular protein, eEF1A2 has potential applications in biotechnology and drug development.^36^ Precise regulation of translation levels affects tumor progression and metastasis. We also examined the role of eEF1A2 K36me3 in the regulation of cell function using a rescue assay. EEF1AKMT4 WT supplementation in EEF1AKMT4 knockdown cells rescued the proliferation, migration, and invasion abilities of GBC cells, whereas EEF1AKMT4 D88A supplementation did not. Meanwhile, compared with eEF1A2 knockdown cells, the proliferation, migration, and invasion abilities of GBC cells were rescued by eEF1A2 WT supplementation but not eEF1A2 K36R supplementation. These results indicate a significant role for K36me3 in eEF1A2 induced GBC progression and lymph node metastasis.

## Conclusions

eEF1A2 is associated with lymph node metastasis and prognosis in patients with gallbladder cancer. eEF1A2 exerts its tumor-promoting effects through the EEF1AKMT4-eEF1A2 K36me3-ribosome protein synthesis-tumor promoting signals axis. eEF1A2 and its K36 trimethylation may be potential prognostic markers and possible therapeutic targets for gallbladder cancer.

## CRediT authorship contribution statement

**Yun-cheng Li:** Resources, Investigation. **Qiang Gao:** Visualization, Validation, Methodology. **Yong-chang Tang:** Resources, Methodology, Data curation. **Zhen-yu Shao:** Formal analysis, Data curation. **Jia-ming Hu:** Resources. **Zeng-li Liu:** Software, Investigation. **An-da Shi:** Visualization, Validation. **Shao-hui Huang:** Investigation, Formal analysis. **Yun-fei Xu:** Supervision, Conceptualization. **Zong-li Zhang:** Writing – review & editing, Supervision, Funding acquisition, Conceptualization. **Kang-shuai Li:** Writing – review & editing, Supervision, Conceptualization.

## Availability of data and materials

All data generated or analyzed during this study are included in this article and its supplementary material files. Further inquiries can be directed to the corresponding author.

## Ethics approval and consent to participate

The study was approved by the Research Ethics Committee of Qilu Hospital of Shandong University. This study was conducted in accordance with the principles of the Declaration of Helsinki and Istanbul.

## Funding information

This work was supported by the 10.13039/501100007129Shandong Province Natural Science Foundation, China (Grant No. ZR2024MH328, ZR2021QH079, ZR2019MH008, ZR2020MH238); Shandong Province Key R&D Program (Major Scientific Innovation Projects), China (2021CXGC011105); 10.13039/501100001809National Natural Science Foundation of China (Grant No. 81900728, 82072676, 82172791, 82203766); Shandong Medical and 10.13039/100018696Health 10.13039/100006180Technology Development Project, China (Grant No. 2018WSB20002); 10.13039/501100009584Clinical Research Foundation of 10.13039/100009108Shandong University, China (Grant No. 2020SDUCRCA018); and 10.13039/100014103Key Research and Development Program of Shandong Province, China (Grant No. 2019GSF108254). The funders had no role in the study design, data collection, analysis, interpretation, or manuscript writing.

## Conflict of interests

The authors declare that they have no competing interests.
