# Supplementary material for: EEF1AKMT4-eEF1A2 synergistically facilitates the progression of GBC by promoting ribosomal protein output
Source: Genes Dis. 2025 Apr 2;13(3):101619. doi: 10.1016/j.gendis.2025.101619 (PMC12907848; doi:10.1016/j.gendis.2025.101619)

**EEF1AKMT4-eEF1A2 synergistically facilitates the progression of GBC by promoting ribosomal protein output**

**Supplemental Materials**

Yun-cheng Li^1, #^, Qiang Gao^1, #^, Yong-chang Tang^#^, Zhen-yu Shao^2, #^, Jia-ming Hu^1^, Zeng-li Liu^3^, An-da Shi^1^, Shao-hui Huang^1^, Yun-fei Xu^1^, Zong-li Zhang^1,*^, Kang-shuai Li^1,*^

^1^Department of General Surgery, Qilu Hospital, Cheeloo College of Medicine, Shandong University, Jinan, Shandong, 250012, China.

^2^Department of Radiotherapy, Qilu Hospital, Cheeloo College of Medicine, Shandong University, Jinan, Shandong, 250012, China;

^3^Department of General Surgery, Qilu Hospital (Qingdao), Cheeloo College of Medicine, Shandong University, Qingdao, Shandong, 266035, China.

**Supplemental Figure 1: Relative expression level of CEACAM5, CD109 and SYT7 compared to eEF1A2 expression in 10 GBC tumor tissues quantified by qPCR.**

**
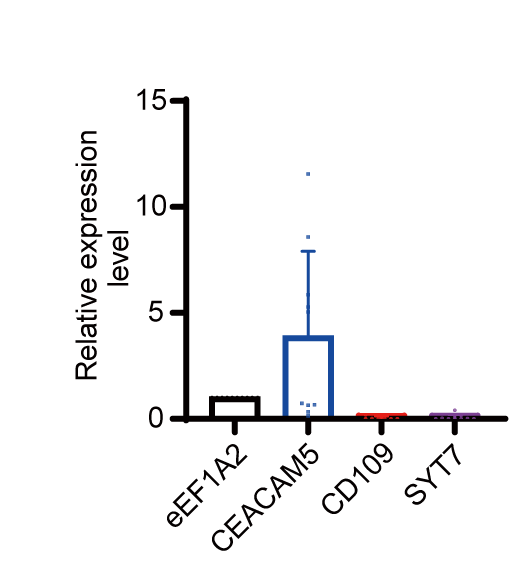
**

**Supplemental Figure 2:** **eEF1A2 exerts oncogenic effects and promotes GBC growth and metastasis.**

A: qPCR analysis of the knockdown efficiency of eEF1A2 in GBCSD and SGC996 cells.

B: qPCR analysis of the effect of eEF1A2 knockdown on the expression of eEF1A1 in GBCSD and SGC996 cells.

C: qPCR analysis of the overexpression efficiency of eEF1A2 in GBCSD and SGC996 cells.

D: qPCR (left) and Western blot (right) analysis of the overexpression efficiency of eEF1A2 in NOZ and OCUG-1 cells.

E: Cell proliferation ability alterations after eEF1A2 overexpression were detected with CCK8 assay in NOZ and OCUG-1 cells.

F: Representative images of clone formation assay of cells with or without eEF1A2 knockdown and overexpression in GBCSD and SGC996 cells.

G: Representative images of wound healing assays to investigate the effect of eEF1A2 knockdown and overexpression on the migration ability of the GBCSD and SGC996 cell lines.

(H-I): Representative images of Transwell assays with matrigel to evaluate the invasive ability of GBCSD and SGC996 cells after eEF1A2 knockdown (H) and overexpression(I).

(J) (left)Representative images of transwell assays with matrigel to evaluate the invasive ability of NOZ and OCUG-1 cells after eEF1A2 overexpression. (right) Statistical analysis of the transwell assays. Overexpression of eEF1A2 promoted the invasion ability of NOZ and OCUG-1 cells.


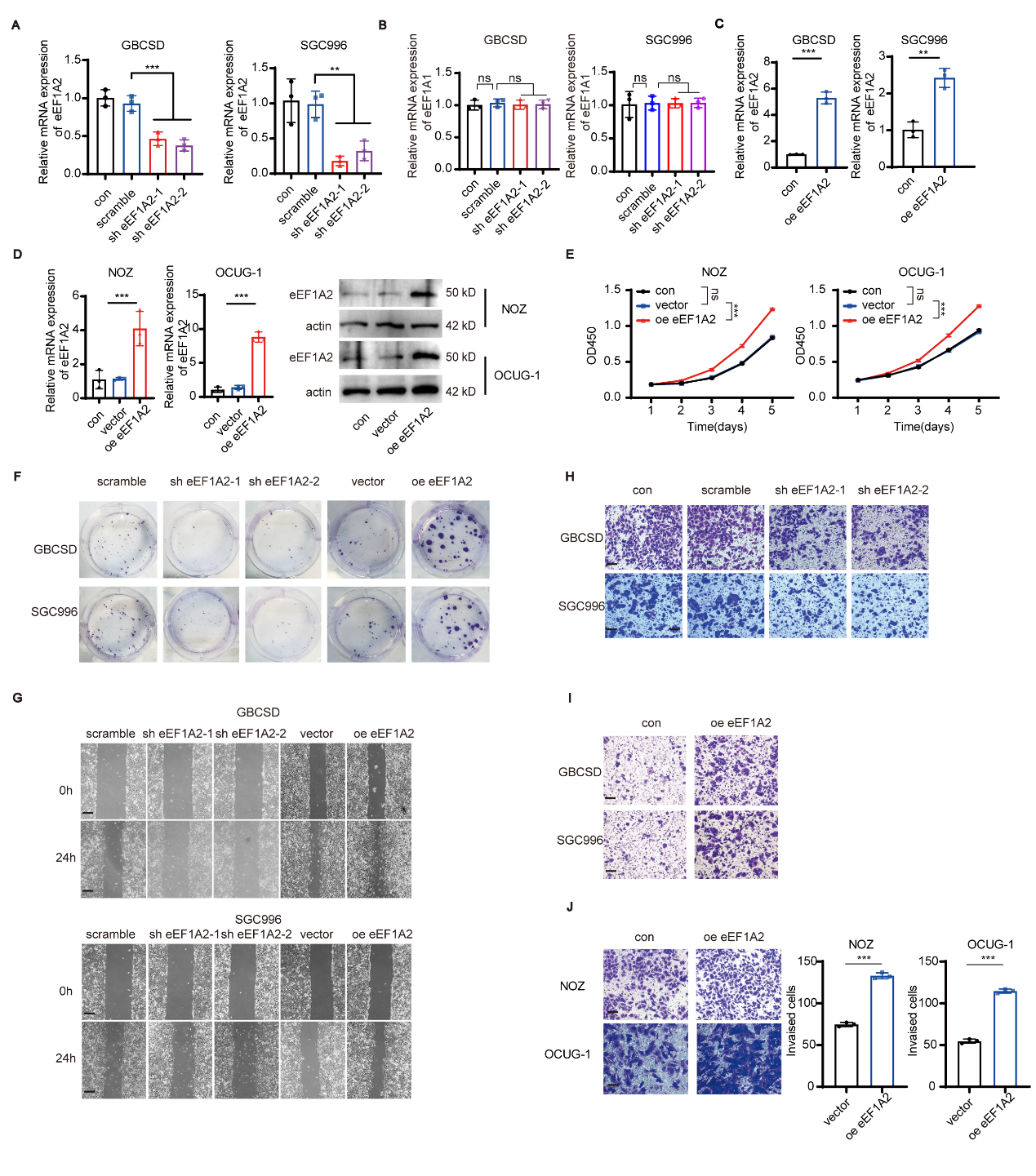


**Supplemental Figure 3:** **Downregulation of METTL13 significantly attenuated the proliferation, migration and invasion of GBC cells in a METTL13-regulated K55 methylation manner.**

A: qPCR analysis of the knockdown efficiency of METTL13 in GBCSD and SGC996 cells. METTL13 was successfully knocked down in both cell lines.

B: Cell proliferation ability alterations after METTL13 downregulation were detected with CCK8 assay in GBCSD and SGC996 cells. Downregulation of METTL13 inhibited the proliferation ability of GBCSD and SGC996 cells.

C: Representative images (left) and statistical analysis (right) of transwell assays with matrigel to evaluate the invasion ability of GBCSD and SGC996 cells after downregulation of METTL13.

D: Representative images (left) and statistical analysis (right) of wound healing assays to investigate the effect of downregulation of METTL13 on the migration ability of the GBCSD and SGC996 cell lines.

**
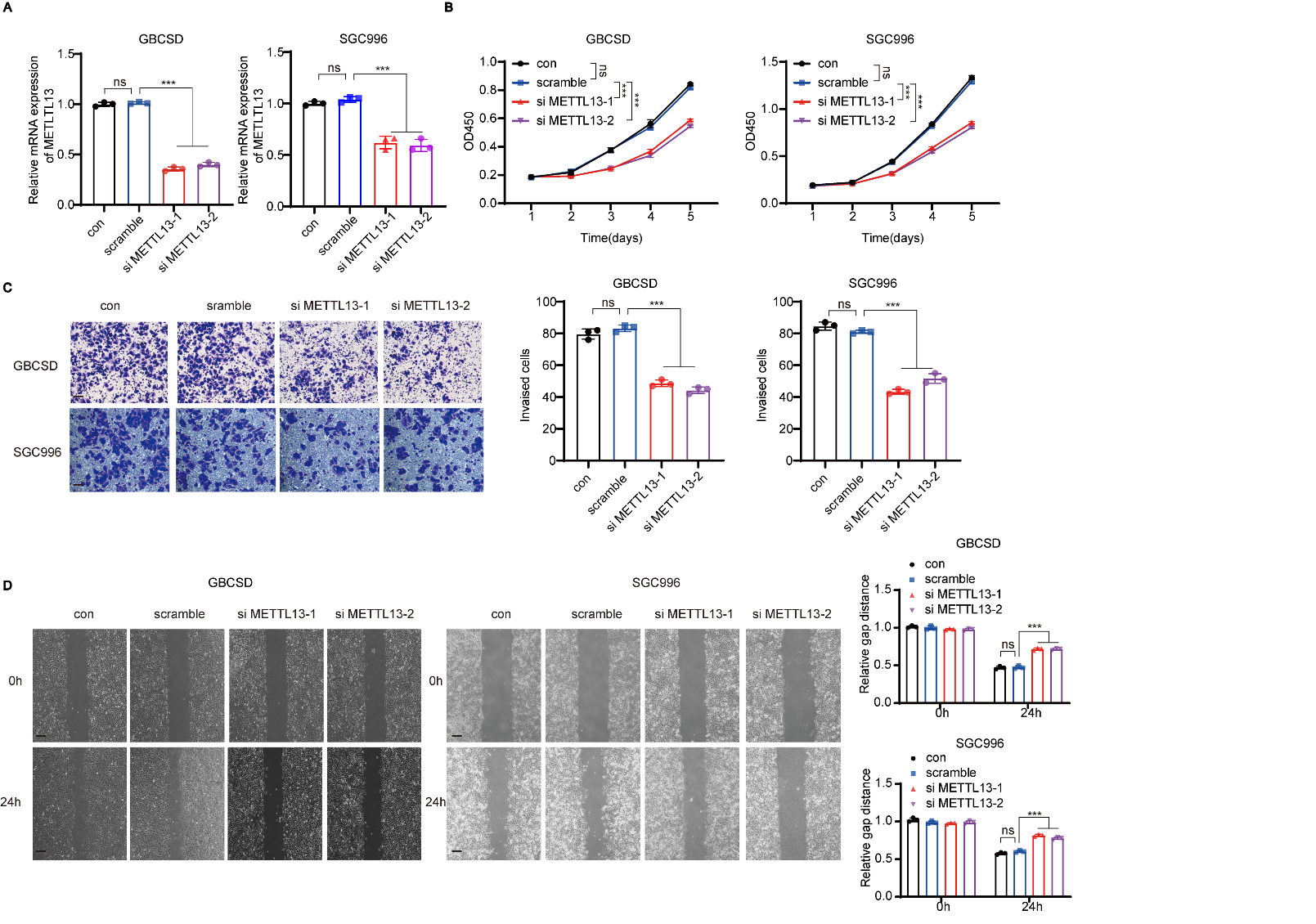
**

**Supplemental Figure 4: Relative mRNA expression level of eEF1A1 in GBC tumor tissues and adjacent normal tissues (*n* = 10).**


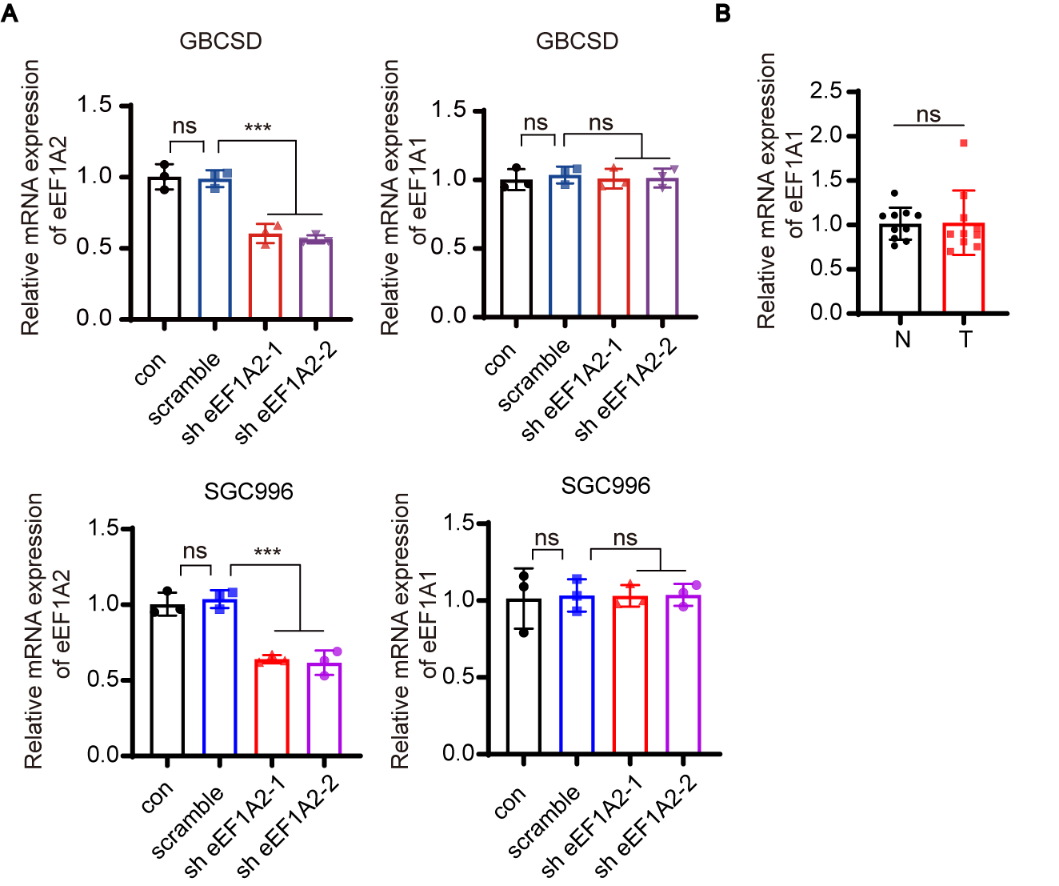


**Supplemental Figure 5:** **Dot blotting assay examining the efficacy and specificity of the anti-eEF1A2K36me3 antibody.**

Dot blotting assay with synthesized eEF1A2K36me0, eEF1A2K36me1, eEF1A2K36me2 and eEF1A2K36me3 peptide (GID（-K(Me3)-）RTIE) was performed to examine the efficacy and specificity of the anti-eEF1A2K36me3 antibody. Anti-eEF1A2K36me3 antibody specifically combined with the eEF1A2K36me3 peptide in a dose dependent manner without combination with the eEF1A2K36me0, eEF1A2K36me1, eEF1A2K36me2 peptide indicating a high specificity of the antibody.


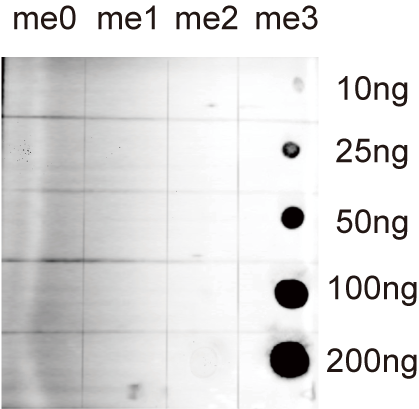


**Supplemental Figure 6:** **Knockdown of EEF1AKMT4 inhibits the malignant phenotype of GBC while its overexpression is not tumor-promoting.**

A: qPCR analysis of the knockdown efficiency of EEF1AKMT4 in GBCSD and SGC996 cells. EEF1AKMT4 was successfully knocked down in both cell lines.

B: qPCR analysis of the overexpression efficiency of EEF1AKMT4 in GBCSD and SGC996 cells. EEF1AKMT4 was successfully overexpressed in both cell lines.

C: Representative images of clone formation assay of cells with EEF1AKMT4 knockdown in GBCSD and SGC996 cells.

D: Representative images of wound healing assays to investigate the effect of EEF1AKMT4 knockdown on the migration ability of the GBCSD and SGC996 cell lines.

(E-F): Representative images of Transwell assays with matrigel to evaluate the invasive ability of GBCSD and SGC996 cells after EEF1AKMT4 knockdown (E) and overexpression(F)

(G): Western blot (left) and statistical analysis (right) of the protein expression levels of EEF1AKMT4 in 6 paired metastatic lymph nodes and primary GBC tumors.


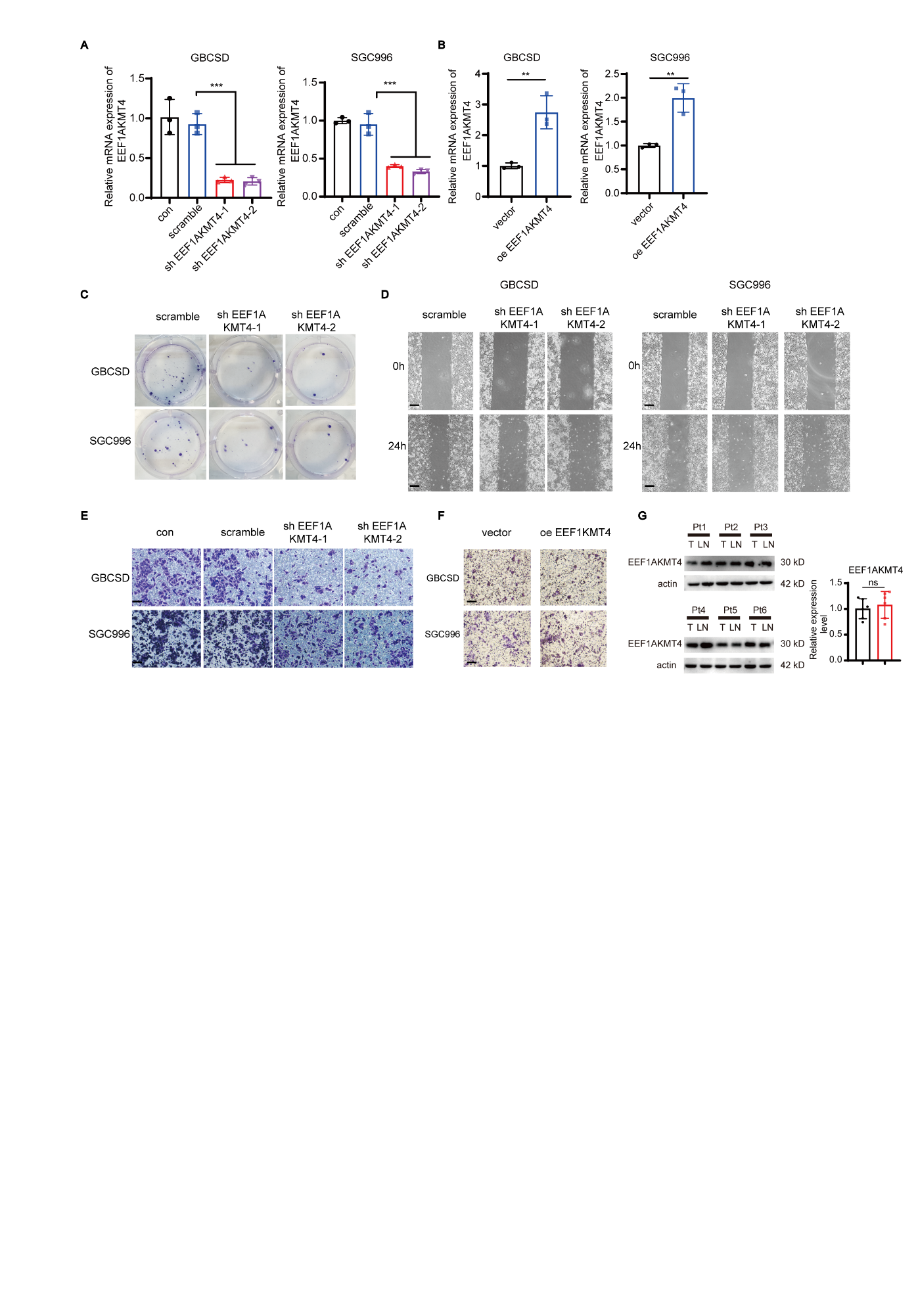


**Supplemental Figure 7: K36 site trimethylation is essential for the tumor-promoting effect of eEF1A2.**

A: Representative images of clone formation assays of cells treated with scrambled shRNA, or eEF1A2-knockdowned GBCSD SGC996 cells complemented with vector, eEF1A2 WT or eEF1A2 K36R.

B: Representative images of wound healing assays of cells treated with scrambled shRNA, or eEF1A2-knockdowned GBCSD SGC996 cells complemented with vector, eEF1A2 WT or eEF1A2 K36R.

C: Representative images of Transwell assays with matrigel of cells treated with scrambled shRNA, or eEF1A2-knockdowned GBCSD SGC996 cells complemented with the vector, eEF1A2 WT, or eEF1A2 K36R.

D: Representative images of clone formation assays of cells treated with scrambled shRNA, or EEF1AKMT4-knockdowned GBCSD SGC996 cells complemented with vector, EEF1AKMT4 WT or EEF1AKMT4 D88A.

E: Representative images of wound healing assays of cells treated with scrambled shRNA, or EEF1AKMT4-knockdowned GBCSD SGC996 cells complemented with vector, EEF1AKMT4 WT or EEF1AKMT4 D88A.

F: Representative images of Transwell assays with matrigel of cells treated with scrambled shRNA, or EEF1AKMT4-knockdowned GBCSD SGC996 cells complemented with the vector, EEF1AKMT4 WT, or EEF1AKMT4 D88A.


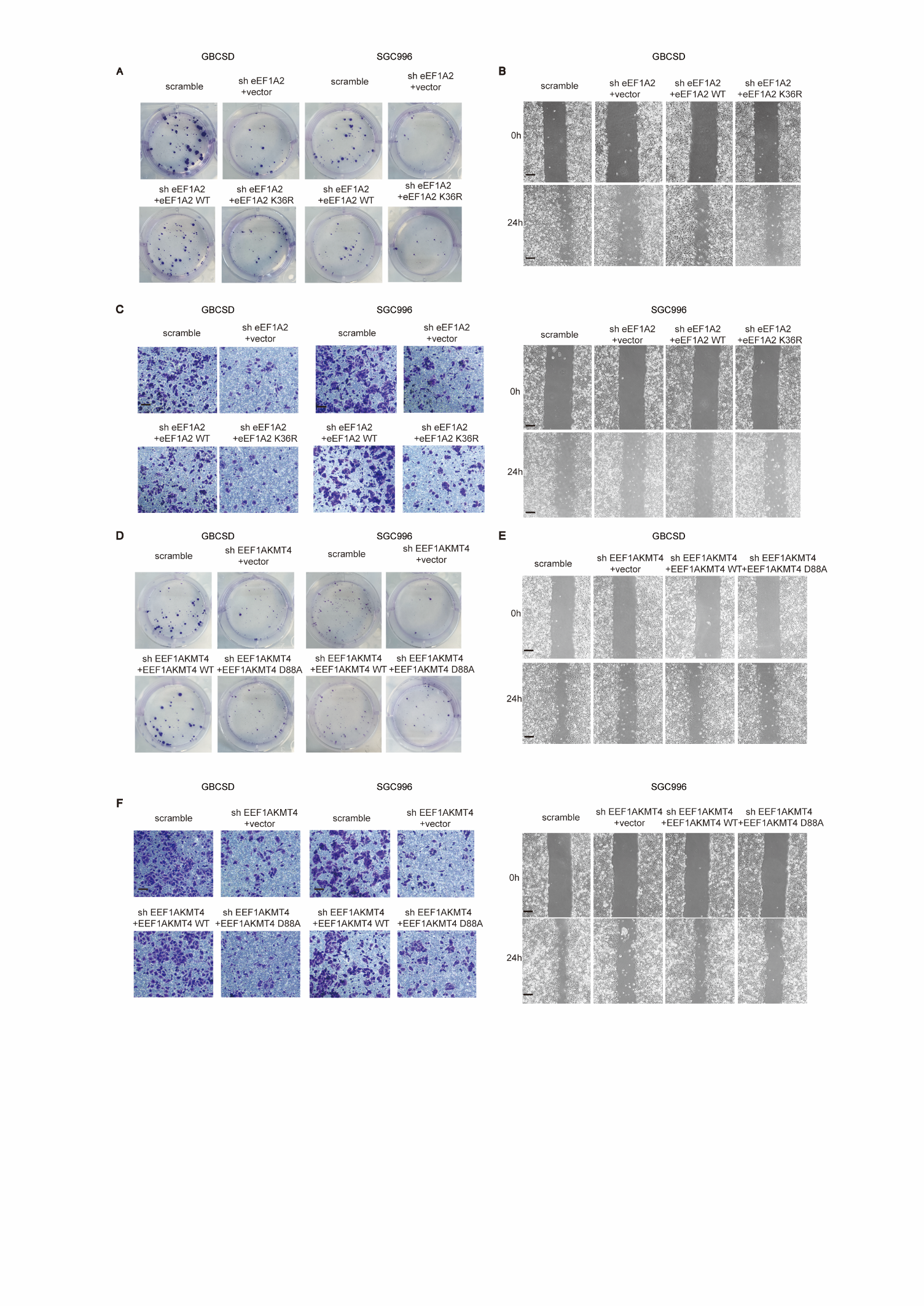

Supplement: Multimedia component 1 [file mmc1.docx]
